# Substitution of human olfaction by the trigeminal system

**DOI:** 10.1126/sciadv.adu7926

**Published:** 2025-11-26

**Authors:** Halina B. Stanley, Clémentine Lipp, Coralie Mignot, Susanne Weise, Konstantinos Garefis, Maxime Fieux, Evangelia Tsakiropoulou, Sotiria Genetzaki, Romain Dubreuil, Camille Ferdenzi, Marina Carulli, Michele Bertolini, Marco Rossoni, Arnaud Bertsch, Juergen Brugger, Thomas Hummel, Iordanis Konstantinidis, Moustafa Bensafi

**Affiliations:** ^1^ Université Claude Bernard Lyon 1, CNRS, INSERM, Centre de Recherche en Neurosciences de Lyon CRNL U1028 UMR5292, NEUROPOP, F-69500 Bron, France.; ^2^ Microsystems Laboratory, École Polytechnique Fédérale de Lausanne (EPFL), 1015 Lausanne, Switzerland.; ^3^ Department of Otorhinolaryngology, Carl Gustav Carus Faculty of Medicine, Dresden University of Technology, Fetscherstrasse 74, 01307 Dresden, Germany.; ^4^ 2nd Academic ORL, Head and Neck Surgery Department, Aristotle University of Thessaloniki, Papageorgiou Hospital, Thessaloniki, Greece.; ^5^ Hospices civils de Lyon, Hôpital Lyon Sud, Service d’ORL, d’otoneurochirurgie et de chirurgie cervico-faciale, F-69310 Pierre Bénite, France.; ^6^ Université de Lyon, Université Lyon 1, F-69003 Lyon, France.; ^7^ Aryballe, 7 rue des arts et métiers, 38000 Grenoble, France.; ^8^ Dipartimento di Meccanica, Politecnico di Milano, Via La Masa 1, Milan, Italy.

## Abstract

Smell loss is a sensory impairment that has major consequences in many areas of daily life and for which current therapies are insufficient. A prosthesis-type technology enabling patients to sample their olfactory environment has not yet been developed. The aim of our study was, therefore, to test whether stimulation of the intranasal trigeminal system by a device combining an artificial nose with an intranasal electrical stimulator will enable patients to detect and discriminate odorant molecules. Four experiments involving normosmic individuals (*n* = 13) and patients with olfactory loss (*n* = 52) showed that individuals were able to detect their olfactory environment using the device. For discrimination, the results are less clear-cut but show that most patients can distinguish between two external stimuli. Although this substitution approach does not allow patients to smell real odors, it is a genuine first substitution solution that we could imagine offering to patients in the future.

## Teaser

The trigeminal system may be used as a substitution sense to detect and discriminate odorants in people with olfactory loss.

## INTRODUCTION

Smells, also known as flavors when present in our food, are both sources of pleasure and social connection (*1*). Smell loss (anosmia, total loss; and hyposmia, partial loss) alters our diet and emotional well-being (*2*, *3*) and increases the risk of domestic accidents (*4*, *5*). Disorders of the sense of smell affect an average of 20% of the population, depending on the type of deficit (anosmia or hyposmia), causes (traumatic, congenital, viral, etc.), age, and pathologies (*6*–*9*). This represents over a billion people worldwide or over a hundred million people in Europe alone. The COVID-19 pandemic also increased the prevalence of postviral smell loss over the past 4 years (*10*, *11*). The economic and social costs of these sensory deficiencies are, therefore, very high. This is why the restoration of olfaction, completely or even partially, is of major importance for science and society.

Restoration of olfaction has been attempted through medication, surgery, and sensory training, but these only work for a limited number of patients; most continue to live without olfaction and face a degraded quality of life (*2*). The development of new technologies to restore olfaction could be a solution. Devices exist for hearing (i.e., cochlear implants) and are now being developed for the vestibular and visual senses. However, there is an unmet need for smell loss as no device (prosthesis or implant) has yet been developed to restore olfaction, despite patient demand (*12*). This is partly because scientific knowledge linking artificial systems to human biological olfaction is still lacking. Artificial devices need to be able to capture odorant molecules in the environment using an artificial system (e-nose) and transform the chemical information into digital and then electrical information, which must then be transmitted to the patient by a stimulation system. While considerable efforts have been made to develop e-noses and map them to human perceptions for the capture part of the device (*13*–*17*), the stimulation part is scientifically much less mature. The few reported studies of electrical stimulation (ES) of the olfactory system through a neurosurgical approach show limited and sometimes paradoxical results (*18*–*22*). While the implantation of cortical electrodes in olfactory zones is likely to evoke olfactory-type percepts (qualities of odors, hedonics, and intensity) in patients (*20*, *23*, *24*) enabling them to recover some perception of odors, and although progress in surgery is increasingly convincing and important (*25*–*27*), it remains a major neurosurgical procedure with risk of complications for patients (e.g., hemorrhage, infection, epileptic seizures, and neurological deficits). Although, in future, the benefit of intracerebral stimulation (recovery of sense of smell and better quality of life) may outweigh the cost of the surgical procedure, at present, we need to devise other paradigms to help patients detect and discriminate their olfactory environment using less invasive devices (*28*). One solution could be to use a substitution system.

Sensory substitution involves using a functional sensory channel to transmit information about another sensory channel that is defective (*29*–*31*). This is a noninvasive technique that leverages the cross-modal plasticity of the brain. Areas of the brain deprived of their normal sensory input (e.g., visual cortex in the blind) become sensitive to alternative input channels (*32*, *33*). Devices enabling such substitution have already been developed to restore visual perception via the tactile (*34*–*36*) or auditory (*37*–*42*) systems. The vOICe device (*43*) uses live camera views and renders the images to soundscapes permitting blind people to obtain three-dimensional information about their surroundings (*44*). A sensory substitution device has also been developed for people with hearing loss (*45*). This device uses a wristband to deliver spatially unique vibrations mapped to phonemes. The brain learns to combine the vibrational information on the wrist with uninterpretable auditory signals to deliver meaningful improvement in speech comprehension. In terms of olfaction, there are as yet no such substitution devices that have been tested in patients with olfactory loss. Here, we investigate whether the intranasal sensitivity of the trigeminal nerve can be used to substitute for defective olfaction. Both the ophthalmic and the maxillary branches of the trigeminal nerve, the fifth cranial nerve, CN-V, carry sensory information from the nasal respiratory epithelium (*46*–*48*) and allow us to perceive two major families of sensation, namely, irritation (ranging from ticklish to tingling to painful) and temperature (coolness and heat). These trigeminal sensations are typically provoked by the interaction of external stimuli with specific trigeminal receptors belonging to the family of transient potential receptors (such as TRPV1 that is activated by capsaicin) (*49*, *50*). Trigeminal fibers can also be stimulated by direct ES of the nasal mucosa, especially within the anterior parts of the nasal cavity (anterior septum, inferior turbinate, and anterior lateral nasal wall), which showed more sensitivity to ES than posterior parts (posterior septum and middle turbinate) (*51*). We believe that stimulating the intranasal trigeminal system is an interesting approach for an olfactory prosthesis as the olfactory and trigeminal systems are anatomically and functionally entwined (*52*), maximizing the chance of triggering olfactory and emotive percepts via trigeminal stimulation.

The general aim of the present study is to assess whether a substitution device using ES of the intranasal trigeminal system can enable humans to detect and discriminate different odorant molecules. We used an artificial nose coupled to an ES system positioned in the nasal cavity. A series of four experiments were carried out on human volunteers with and without olfactory disorders. These experiments were carried out under the same conditions in three different labs (Lyon, France; Thessaloniki, Greece; and Dresden, Germany) collaborating in a joint effort. In these experiments, different odorant molecules were presented at the input of the system, and, for each stimulus, the participants received a specific ES pattern. The participants’ task was to detect the stimulus and discriminate between the stimuli. The specific aim of the study was to test the hypothesis that the trigeminal system will transduce the electrical pattern signal into percepts and that these sensations will enable participants to (i) detect odorants and (ii) discriminate between odorant molecules.

## RESULTS

### Combining an artificial nose and a nasal stimulator

**Fig. 1.**
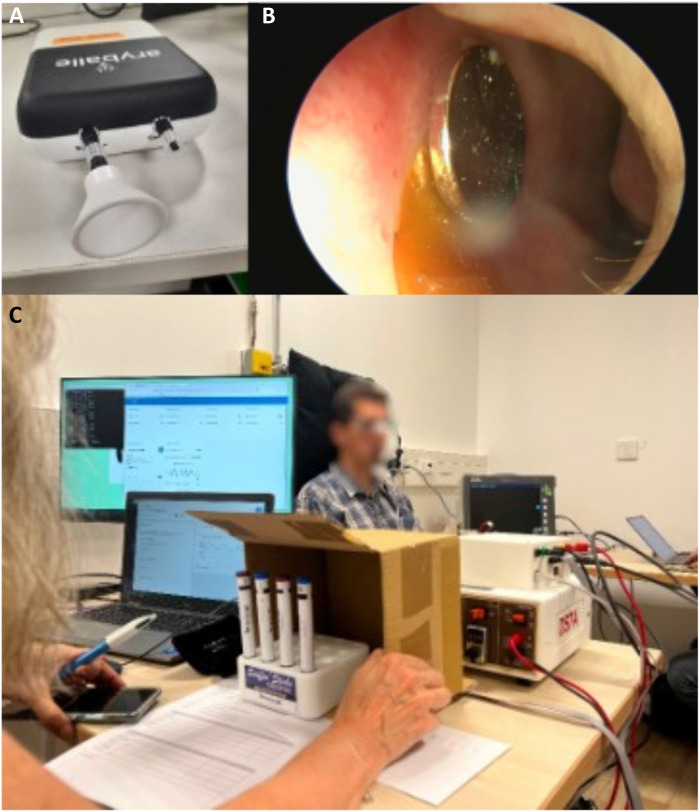
The experimental device combining the artificial nose and the stimulator. The device consists of a VOC detector (**A**) coupled to the clip style stimulator (**B**) via a custom interface (**C**).

The first stage of the present study was to set up the device combining artificial sensors and a stimulator (Fig. 1). The olfactory sensor network (Fig. 1A) consists of an optical sensor (Aryballe Technologie’s Neose-Advance device) that can detect and discriminate between several categories of volatile organic compounds (four odorant molecules in this study). The odorants are presented in Sniffin Sticks format at the entrance to the artificial nose. An algorithm classifies the odorant on the basis of its fingerprint in real time, and a graphical user interface associates this fingerprint with a specific ES pattern. This ES pattern is sent via a magnetic clip stimulator (placed on the nasal septum, Fig. 1B), which maintains good contact between the electrodes and the nasal mucosa. Figure 1C shows the operational device. Further details are provided in the Supplementary Materials (Supplementary Text S1 and S2).

### Experiment 1

**Fig. 2.**
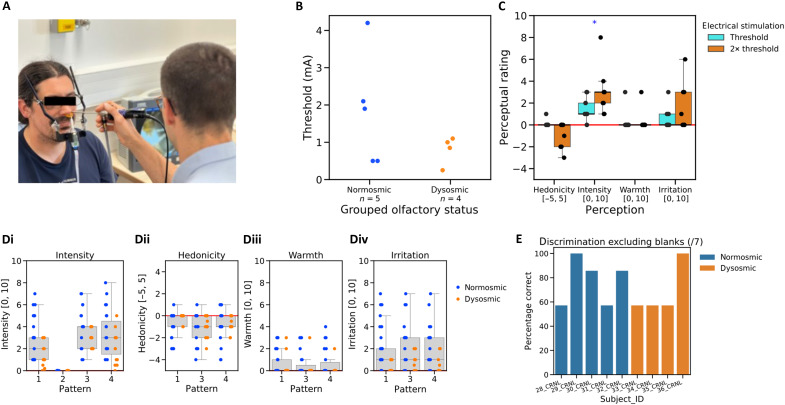
Psychophysical responses in experiment 1. (**A**) Stimulation device on a participant and ENT examination. (**B**) Threshold for ES at the septum. (**C**) Perception of the ES at threshold and twice threshold (single stimulation of duration, 500 μs). (**D**) Participant’s subjective perception of intensity (i), hedonicity (ii), warmth (iii), and irritation (iv) for the different stimulation patterns. For detection, stimulation pattern #2 was the control (no stimulation), and no participant reported feeling anything. (**E**) Performance on the discrimination task for all nine participants. *P ≤ 0.05.

Once the device was operational (Figs. 1C and 2A), we launched a first exploratory experiment (experiment 1). Experiment 1 tested the hypothesis that human volunteers, with or without a sense of smell, can detect and discriminate odorous molecules using the device described above. Nine volunteers were included in the study (five normosmic participants and four patients with olfactory disorder) (see Materials and Methods and Supplementary Text S3). Figure 2B shows the threshold values for the nine volunteers, and Fig. 2C depicts perceptual responses for a single ES at the threshold and at twice the threshold current. It shows that the threshold for ES at the septum is heterogeneous but independent of olfactory status. Moreover, increasing the current from the threshold to twice the threshold caused an increase in perceived intensity (*W =* 0, *P* = 0.021, double sided), but there were no statistically significant differences for any of the other perceptions (Fig. 2C).

After the baseline response to ES had been established, the participants received a sequence of ES patterns. These patterns were identical in terms of duration of the electrical pulses (500 μs) and their intensity (current set at twice threshold) but differed in the number of electrical pulses per pattern and the time between pulses. Four patterns were set up, one of which was a dummy (see Materials and Methods and Supplementary Text S3). Participants were asked to rate their perception of each ES pattern in terms of its intensity, hedonicity, warmth, and irritation. They also had to decide, except for the first in the series, whether the ES pattern was the same as, or different from, the one previously presented. The sequence included four pairs comparing the dummy with a real ES pattern and seven pairs comparing different ES patterns. Comparing the dummy stimulation (#2) with the three real stimulation patterns [Fig. 2D (i)], we see a clear difference in intensity [χ^2^(3) *=* 44.9, *P <* 0.00001]. The control pattern #2 was rated significantly lower intensity than the three other patterns (*P <* 0.00001 in all pairwise comparisons). All of the nine volunteers gave a zero-intensity rating to the control stimulus (#2, no ES), and only one gave a zero-intensity rating to any real ES [Fig. 2D (i)]. The volunteers were, therefore, able to detect ES compared with no stimulation. All sequences of stimulations (#1, #3, and #4) were found to be moderately intense, slightly unpleasant, and slightly irritating [Fig. 2D, (i), (ii), and (iv)]. There was a very slight perception of warmth [Fig. 2D (iii)]. There were no statistically significant differences between the different sequences of stimulations other than stimulation pattern #3 rated more intense than pattern #1 [*P =* 0.02236, Fig. 2D (i)].

Then, we assessed whether participants could determine whether consecutive real ES patterns were the same or different (the same/different task). In our sequence, ignoring the dummy stimulations, we have four pairs that are different (1-3, 1-4, 4-3, and 4-1) and three pairs that are the same (1-1, 3-3, and 4-4) (see Supplementary Text S3). Four of the nine participants were able to determine which of the seven pairs in the series of electrical stimuli were the same and which ones were different (two normosmics with 86% correct responses, one normosmic with 100% correct responses, and one anosmic with 100% correct responses), while, for the other five participants, their success rate was no better than chance (Fig. 2E). In sum, this first experiment suggests that participants with olfactory loss are able to detect patterns of ES that were coupled with olfactory stimuli detected by an artificial nose, but that discrimination may be more difficult.

Experiment 1 did, however, have some limitations. Participants were not given precise information about the intranasal electrical sensation that they should expect, which may have impeded their ability to discriminate between stimulation patterns. Moreover, according to verbal feedback, it is not unlikely that participants were unsure of what was meant by “same/different” in the task of comparing successive stimuli. We also note the difficulty of the task. By integrating two tasks to be performed at the same time (same/different and perceptual judgments including detection) in the same stimulation series, the cognitive effort required of the participants was higher than if the two tasks had been separated into two distinct blocks and the low success rate of some participants may simply arise from cognitive overload. Last, the support system associated with the stimulator made the device quite heavy (Fig. 2A). This caused increasing discomfort at the bridge of the nose as the experimental session progressed, which may have disrupted the perception of the stimulation and reduced the participants’ attention span.

### Experiment 2

**Fig. 3.**
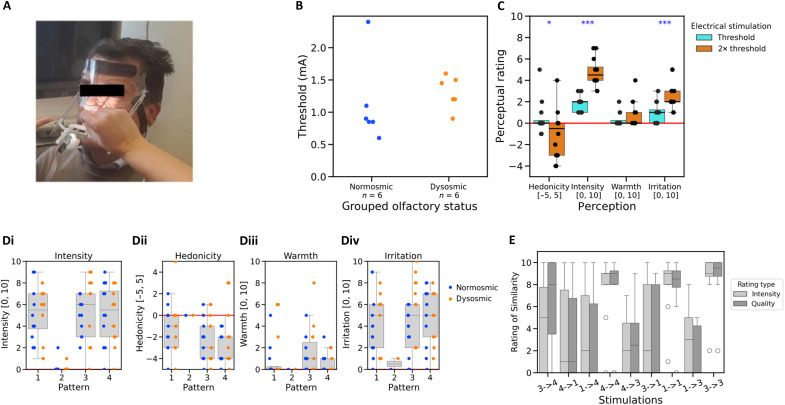
Psychophysical responses in experiment 2. (**A**) New version of the stimulation device. (**B**) Threshold for ES at the septum. (**C**) Perception of the ES at threshold and twice threshold (single stimulation of duration, 500 μs). (**D**) Participant’s subjective perception of intensity (i), hedonicity (ii), warmth (iii), and irritation (iv) for the different stimulation patterns. (**E**) Performance on the discrimination task for identical (1-1, 3-3, and 4-4) and different (1-3, 3-1, 1-4, 4-1, 3-4, and 4-3) pairs. *P ≤ 0.05; ***P < 0.001.

To take all these limitations into account, we ran a second experiment (experiment 2) in 12 volunteers (six normosmics and six patients with olfactory disorders) (see Materials and Methods and Supplementary Text S4). Here, the detection task was separated from the same/different task. We also asked for further clarification of similarity or difference by using the two dimensions of intensity and perceptual quality and introduced a new stimulator support device (Fig. 3A). As for experiment 1, the detection threshold for ES at the septum is heterogeneous but independent of olfactory status (Fig. 3B). Moreover, increasing the current from threshold to twice threshold caused an increase in intensity (*W* = 0, *P =* 0.00236) and an increase in irritation (*W* = 0, *P* = 0.00203) and a decrease in hedonic valence (*W* = 43, *P* = 0.01644) (Fig. 3C) but no difference in warmth (*W* = 2, *P* = 0.16755).

Regarding the detection task, Fig. 3D illustrates the participant’s ratings of intensity, hedonicity, warmth, and irritation for the four stimulation patterns. For perceived intensity, as in experiment 1, we observed an effect of stimulus pattern on intensity ratings [χ^2^(3) = 113, *P* < 0.00001] [Fig. 3D (i)]. Here, the control pattern #2 was significantly less intense than the three other patterns (*P* < 0.00001 in all cases). There was no significant difference in intensity between patterns #1, #3, and #4 (*P* > 0.050 in all cases). Stimulation detection was excellent; there were only two (of 72) occasions when a participant gave a nonzero intensity for the control stimulus (pattern #2). Conversely for the real stimulations, pattern #1 was never given a rating of zero intensity, pattern #3 only once, and pattern #4 twice (8.3%). Overall, all real stimulation patterns (#1, #3, and #4) were perceived as moderately intense [Fig. 3D (i)], slightly unpleasant [Fig. 3D (ii)], and irritating [Fig. 3D (iv)]. Perception of warmth was absent for most participants [Fig. 3D (iii)].

Regarding the discrimination task, on a descriptive level, Fig. 3E shows that, whereas the three identical pairs (#4-#4, #1-#1, and #3-#3) were rated as very similar in terms of intensity and quality, the different pairs were on average rated as less similar. A statistical analysis comparing the similarity ratings of identical versus different pairs revealed a significantly higher similarity in terms of intensity (*W* = 77, *P* = 0.0032) and quality (*W* = 75, *P* = 0.0053) for the identical pairs. Furthermore, this analysis showed no effect of olfactory status on intensity (same pairs: Mann-Whitney *U* = 11, *P* = 0.293; different pairs: *U* = 18, *P* = 1.00) and quality (same pairs: Mann-Whitney *U* = 14.5, *P* = 0.625; different pairs: *U* = 11.5, *P* = 0.332) similarity judgements, suggesting that this discrimination ability is observed equally in normosmics and patients with olfactory deficits.

### Experiment 3

**Fig. 4.**
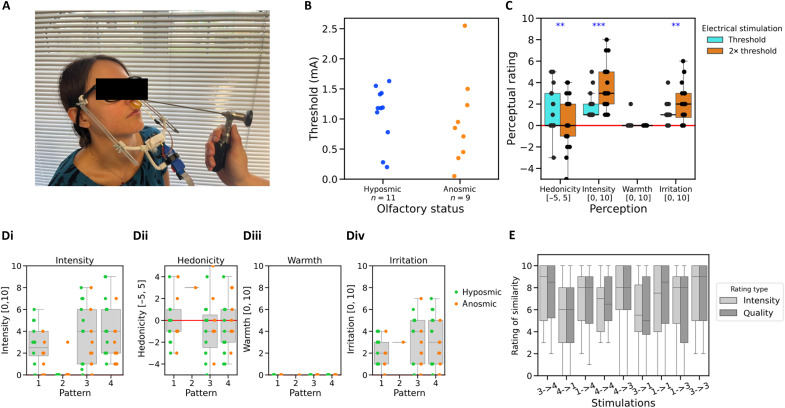
Psychophysical responses in experiment 3. (**A**) Stimulation device and ENT examination. (**B**) Threshold for ES at the septum. (**C**) Perception of the ES at threshold and twice threshold (single stimulation of duration, 500 μs). (**D**) Participant’s subjective perception of intensity (i), hedonicity (ii), warmth (iii), and irritation (iv) for the different stimulation patterns. (**E**) Performances on the discrimination task for identical (1-1, 3-3, and 4-4) and different (1-3, 3-1, 1-4, 4-1, 3-4, and 4-3) pairs. **P < 0.01; ***P < 0.001.

In a third experiment (experiment 3), we set out to replicate the results of experiment 2 on a larger sample, considering only patients with olfactory disorders. To simplify the experiment, we used the stimulator from experiment 1 (Fig. 4A), but without the artificial nose. We have results for 20 volunteers (9 anosmics and 11 hyposmics) (see Materials and Methods and Supplementary Text S5). As in experiments 1 and 2, one can see that the threshold for ES at the septum is heterogeneous but independent of the etiology of the olfactory deficit (Fig. 4B). Moreover, increasing the current from threshold to twice threshold, participants report an increase in intensity (*W* = 0, *P* = 0.00041) and irritation (*W* = 9, *P* = 0.00571) and a decrease in hedonicity (*W* = 78, *P* = 0.00199) (Fig. 4C). The perception of warmth was essentially absent.

Figure 4D shows the participant’s ratings of intensity, hedonicity, warmth, and irritation for the four different stimulation patterns. For perceived intensity [Fig. 4D (i)], an effect of stimulus pattern on intensity ratings was observed [χ^2^(3) *=* 81.8, *P <* 0.00001]; the control pattern #2 was significantly less intense than the three other patterns (*P* < 0.00001 in all cases); there were no differences in intensity between the three other stimulation patterns (*P* > 0.050 in all cases). As in experiments 1 and 2, ES detection was good: There was only one instance of a nonzero rating of intensity for the dummy ES pattern #2 (1.7%) [Fig. 4D (i)]. All real stimulation patterns (#1, #3, and #4) were perceived as moderately intense [Fig. 4D (i)], moderately unpleasant [Fig. 4D (ii)], and irritating [Fig. 4D (iv)]. There was no perception of warmth [Fig. 4D (iii)]. However, in contrast to experiment 2, the results were much less convincing concerning the participants’ ability to distinguish between different patterns of ES (same/different task) (Fig. 4E). Statistical analysis comparing similarity judgements between identical and different pairs revealed no differences between pair types in terms of intensity (*W* = 145, *P* = 0.145) or quality (*W* = 134, *P* = 0.121).

In summary, from the first three experiments, we find that all the participants in our study, irrespective of olfactory function, have very good ability to detect ES that could be used to signal the presence of an odor in their environment. The second result is that, when it comes to distinguishing between different patterns of stimulation, performance is lower and experiment 3 does not reproduce the results of experiment 2. One notable difference between experiments 2 and 3, however, is that, for simplicity reasons, experiment 3 used only the stimulation device, without the artificial nose. Consequently, participants did not receive the (e-nose–driven) auditory preparatory countdown before each stimulation that participants in experiment 2 received. It is possible that this lack of warning before the stimulation made the discrimination task more difficult. In any case, the cognitive effort required to rate a series of 10 successive stimulations is high, which may have limited performance. Last, participants were not familiarized with the ES patterns that may have impeded their ability to discriminate between stimulation patterns.

### Experiment 4

We, therefore, designed experiment 4 in which we familiarized the participants with the stimulation patterns, included an auditory countdown warning before each stimulation, and proposed a simplified discrimination task (see Materials and Methods and Supplementary Text S6). To reduce the experiment duration, we opted to include only two stimulation patterns instead of three, making the experiment acceptable for the participant in terms of duration and potential fatigue. Stimulation patterns #1 and #3 (as described before) were selected on the basis of a meta-analysis of the perceptual data collected in experiments 1 to 3 (see Supplementary Text S7). The discrimination task consisted of choosing the “odd-one-out” from a set of three stimulations: two the same and one different.

**Fig. 5.**
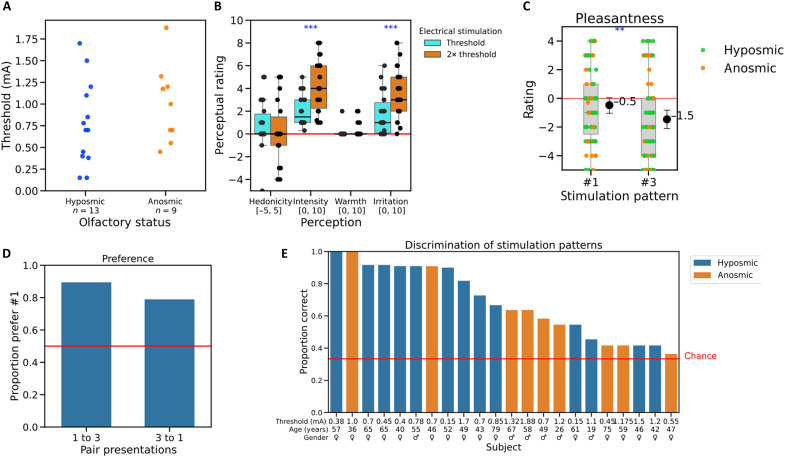
Psychophysical responses in experiment 4. (**A**) Threshold for ES at the septum. (**B**) Perception of the ES at threshold and twice threshold. (**C**) Ratings of hedonic valence of the two stimulation patterns. (**D**) Preference for pattern #1 versus #3. (**E**) Performance on the discrimination task. **P < 0.01; ***P < 0.001.

A total of 22 volunteers were included in experiment 4 (9 anosmic and 13 hyposmic). The data show that the threshold for ES at the septum is heterogeneous but independent of type olfactory deficit (hyposmia or anosmia) (Fig. 5A). Furthermore, as in experiments 1 to 3, on increasing the threshold current to twice the threshold, patients reported an increase in intensity (*W* = 0, *P* = 0.00008) and irritation (*W* = 13, *P* = 0.00052) (Fig. 5B).

**Fig. 6.**
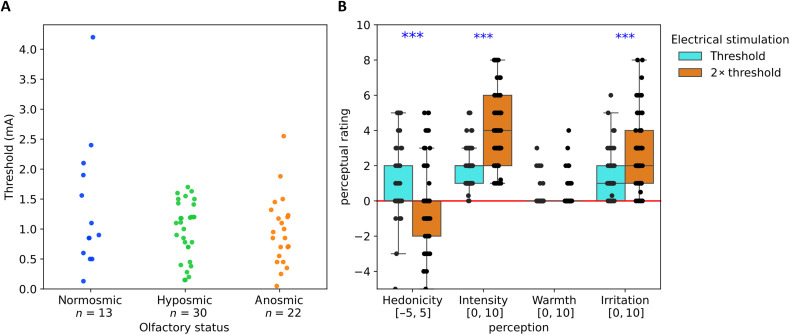
Detection thresholds and psychophysical responses from baseline measurements from combined data from experiments 1 to 4. (**A**) Threshold for ES at the septum. (**B**) Perception of the ES at threshold and twice threshold. ***P < 0.001.

It should be noted here that the protocol carried out for thresholds and perceptual evaluations of threshold and double-threshold values is the same across the four experiments. To gain in statistical power, we, therefore, conducted a meta-analysis combining data from these four experiments (data from 13 normosmic, 30 hyposmic, and 22 anosmic participants). A one-way nonparametric analysis of variance (ANOVA) on the threshold data by olfactory status (Kruskal-Wallis test) shows no difference in the threshold for detection of ES [χ^2^(2) = 0.759, *P* = 0.648, ε^2^ = 0.012]. Pairwise comparisons also show no differences (normosmic-hyposmic: *W* = −0.861, *P* = 0.8155; normosmic-anosmic: *W* = −1.136, *P* = 0.7012; hyposmic-anosmic: *W* = −0.642, *P* = 0.8928) (Fig. 6A). Paired sample *t* tests comparing the perceptions at threshold with those at double-threshold intensity (Fig. 6B) show that the hedonicity at double threshold is lower than at threshold (*W* = 694, *P* = 0.00002) and both intensity and irritation are perceived to be higher at double threshold than at threshold (*W* = 0, *P* < 0.00001 and *W* = 76.5, *P* < 0.00001, respectively). There was no difference in perception of warmth, which was essentially absent (the 85th percentile was zero at threshold and 1 at double-threshold current).

Returning to experiment 4, during the familiarization task with the two ES patterns, participants were asked to rate their hedonic value and give their preference for one of the two patterns. Analysis of the data shows that pattern #3 was significantly more unpleasant than pattern #1 (Mann-Whitney *U* = 2682, *P* = 0.0095; Fig. 5C), a result in line with the preference data between the two patterns, which shows a preference for pattern #1 versus pattern #3 (80.5%: 66 of the 82 answers from 21 participants, no answers recorded from one participant; Fig. 5D). Last, the results of the discrimination task showed that most participants could determine the odd-one-out ES with a good degree of success; overall performance was significantly better than chance (*W* = 252, *P* < 0.0001, *H_a_* μ > 0.3333). Two participants were correct 100% of the time, and 16 of the 22 participants were correct more than 50% of the time (Fig. 5E). There was no difference in performance between the hyposmic and anosmic participants (*t*_20_*=* −1.35, *P* = 0.19335).

## DISCUSSION

The present study is part of a broader theme of the rehabilitation of olfactory deficits (*9*). These deficits affect around 20% of the world’s population (*6*, *53*), and the techniques used to help patients are based around three areas: medication, surgery, and olfactory training (*53*). To date, there is no technology that can be used as a prosthesis or neural implant for the sense of smell (*11*, *12*, *54*). It has been shown that it is possible to replace some visual perception via tactile (*55*, *56*) or auditory (*38*, *41*, *42*, *57*) channels and hearing via touch (*58*, *59*). Our study investigates the possibility that the trigeminal function, via its intranasal branch, may be co-opted in a similar way for olfactory loss.

Based on the assumption that ES of the nasal cavity can recruit intranasal sensory fibers of the trigeminal nerve and convey sensory and emotional information to the participant, a four-step experiment process was designed to see whether patients can associate this ES with a given odor.

A device combining a set of artificial olfactory sensors and a neural stimulator was developed and tested in patients with smell disorders. The odorant molecules presented to the sensors were detected, and the chemical information was transformed into digital information, which, in turn, was associated with a specific stimulation pattern. In this way, each olfactory stimulus was associated with a specific ES. In the first three experiments of our study, we showed that individuals (normosmic or with partial/severe olfactory loss) were able to detect the ES. This initial result is of great clinical interest, as it makes it possible to imagine restoring the ability to detect the olfactory environment in a person who does not have any sense of smell. From our meta-analysis of the baseline data (Fig. 6), we observed that the patients’ sensitivity to ES was not significantly different to that of normosmics.

Our results are less robust concerning discrimination abilities of ES and, hence, the information capacity available via this channel. In the first experiment, around half the participants performed very well, while the others did no better than chance. On the basis of these results, we changed the discrimination protocol for experiments 2 and 3 to reduce cognitive load, separate differences in intensity from qualitative differences (number of pulses), and provide participants with a difference scale rather than a simple binary choice. While the results of experiment 2 were encouraging, experiment 3 did not reproduce them even though the protocols were similar. We, therefore, set up experiment 4, in which the participants were first briefly familiarized with the stimulation patterns and then given a simplified discrimination task which eliminated any bias introduced by our sequence of pairs. In this experiment, more than two-thirds of the participants had good or very good discrimination skills, and there was no significant difference in performance between the hyposmic (*n* = 13) and anosmic (*n* = 9) patients. These findings suggest that it is possible for patients to use intranasal ES patterns to receive olfactory information.

In this study, odors were matched to stimulations arbitrarily, and the association between odor and stimulation was not explored. In future, we intend to exploit an associative learning paradigm. Studies have already shown cross-modal associative learning of emotionally salient odors (*60*, *61*). Although our participants were anosmic and unable to perceive odors directly, we anticipate that leveraging perceivable and discriminable trigeminal stimulation will allow them to form associations through mental imagery. This approach may clarify whether anosmics can establish robust cross-modal links despite their olfactory deficit.

Olfactory space is complex, and humans detect a vast number of different odors and olfactory qualities (*62*, *63*), whereas the trigeminal system provides a much smaller number of different perceptions. Substituting olfaction by trigeminal stimulation may, therefore, be limited by information transfer. This study was confined to examining three different stimulation patterns and a blank (two patterns for experiment 4). A device that is to be useful for patients will need to provide more information. Our experiments restricted stimulation to simple patterns of ES in one nostril and with fixed intensity. One might imagine bilateral stimulation (changing the position of stimulation), variable intensity stimulations, and the addition of other modality stimulations [e.g., mechanical and/or thermal stimulations to which the trigeminal nerve is also sensitive; (*64*)]. The degree to which patients can discriminate between different trigeminal stimulations, and, therefore, the maximum possible information content of the signals, is, however, an open question.

To improve performance an obvious avenue to explore is, of course, training. The brain’s plasticity with respect to training is well known (*65*, *66*) as is cross-modal plasticity in the cases of blind and deaf individuals (*67*). Less is known specifically about anosmic patients, although olfactory training is used as a therapy for anosmia and shows effects at both peripheral and central levels (*68*, *69*). Hummel *et al*.(*70*) showed that patients who had lost their sense of smell could recover certain olfactory abilities following repeated exposure to odors over 3 months, and Fournel *et al*. (*71*) showed that familiarization with odors associated with verbal labels for three sessions over 1 week could increase the ability to name odors with functional modulation of associative brain regions. We can, therefore, hypothesize that a training regime designed along the same lines as those used for other areas of neurohabilitation (*72*) should be beneficial for improving patients’ discrimination and recognition abilities. We note that biological olfaction relies on respiration transporting odorants present in the environment into the nasal cavity and olfactory receptors. The sniff is part of the olfactory percept (*73*), and its active use will encourage the development of true sensory substitution. Although it is technically not feasible today to place odor sensors inside the nasal cavity, it would be possible to implement a sensor of respiration to trigger olfactory input information, thus mimicking natural sniffing behavior.

However, trigeminal stimulation alone may not be sufficient to compensate for the loss of smell. Possible developments of our method could, thus, include, in addition to trigeminal stimulation, the stimulation of other senses such as sight or hearing to complement the ES and enrich the percept generated in the patient. These avenues should be explored in future developments.

This raises the question of whether the choice of the trigeminal system for sensory substitution is ideal. Possible substitution routes include the visual, auditory, tactile, and gustatory systems. However, each of these pathways presents problems. First, the emotional experience is different for vision, hearing, and olfaction. Second, it is advantageous for the substitution system to be linked to human respiration in the same way as the sense of smell, but none of these four modalities allow odorant molecules to be sampled via sniffing. In contrast, both olfactory and trigeminal receptors are located in the nasal cavity and both systems are accompanied by a motor action that ranges from simple passive nasal inhalation to active exploratory sniffing. There are several arguments in favor of using the trigeminal system as a substitute for the sense of smell. First, olfactory and trigeminal systems are closely interlinked both anatomically and functionally. Anatomically, there are collaterals of the trigeminal nerve that end within the olfactory epithelium; some even reenter the central nervous system and terminate within the olfactory bulb (*74*–*76*). Functionally, recruitment of intranasal trigeminal receptors induces neural activation in both somatosensory areas and primary olfactory areas (*77*), and most odors activate the olfactory and trigeminal systems simultaneously (*52*, *78*). Second, as said above, breathing is of great importance for the functioning of both olfactory and trigeminal systems (*49*, *73*). The two systems work together to form the watchdog of the nose. In synergy with the sense of smell, the trigeminal system can prevent the inhalation of potentially life-threatening substances by stopping the inspiration. Third, the trigeminal nerve permits lateralization. A preliminary study in four participants suggests that intranasal ES of the trigeminal system may allow an odor to be localized (*79*). Fourth, the intranasal trigeminal system and the olfactory system often interact to produce affective responses to chemical stimulations (hedonic valence): Most odors are described using not just olfactory but also trigeminal verbal descriptors [smells can be described as painful and cold; (*80*)], and functional magnetic resonance imaging studies showed that such trigeminal sensations are mediated by an emotional area, the amygdala (*81*), highlighting the ability of trigeminal stimulation to generate emotions.

We also need to consider whether patients would be interested in such an intranasal device. In an initial study, Besser *et al*. (*12*) showed that 30% of patients who had lost their sense of smell were in favor of an olfactory implant solution. These results were confirmed by a study by (*11*), which showed that a quarter of patients would accept an option similar to the one used in the present study, i.e., a technology with minimal invasiveness (the stimulation system used in our study is a magnetic clip that can be removed in less than a second by the patient simply pulling it out of his nose). Furthermore, Pinger *et al*. (*54*) showed that 60% of patients suffering from loss of smell were interested in olfactory technologies as a therapeutic option. Patients anticipated greater benefits in detecting danger and assessing the edibility of food. This suggests that initial efforts should focus on the primary functions of olfaction, particularly hazard detection and the distinction between spoiled and edible foods. These two primary functions of the sense of smell can largely be covered by the method used in our study by associating one family of stimulation patterns with a specific category of odors and another family of stimulation patterns with another olfactory category. For example, the unpleasant nature of ES pattern #3 could be used to signal inedible and/or unpleasant odors with pattern #1 used for a less unpleasant category of odor. Such technology can be expected to have a direct and perceptible effect on the people affected. Last, we note that our device is modular; the stimulator could be used with different odor detectors, and the odor detector could be used with an intracerebral stimulator.

We list several limitations and potential points for improvement to be incorporated into future studies. First, we worked on a very small panel of odorants (four different stimuli and their associated stimulation patterns), and it will be necessary in future experiments to integrate a greater diversity of olfactory compounds. Consequently, in terms of stimulation, we will have to introduce a larger number and wider spectrum of stimulation, ideally covering extreme hedonic valences ranging from unpleasant to pleasant, whereas our current stimulations are all mildly unpleasant. We can hypothesize that, with training, certain familiar stimuli will become pleasant, in accordance with the mere exposure effect (*82*). In addition, it will also be possible to vary the stimulation parameters (amplitude, frequency, and duration) to increase the differences in perception, as well as incorporating bilateral stimulation or changing nostrils, which should improve discrimination. Second, although we have some data suggesting patient interest in such a device, it would be useful to test acceptability of different designs further with patients. This could be done in the form of workshops with nonfunctional prototypes that the patients can wear and imagine using. Third, our device (Fig. 1) is not wearable so a second axis of R&D concerns the ergonomics of the system. It will need to be miniaturized to make it accessible for experimental study in a living lab, hospital, or home to assess its usefulness in a context that is relevant for the person. Last, we tested the device on a relatively small number of volunteers: 9 in experiment 1, 12 in experiment 2, 20 in experiment 3, and 22 in experiment 4. Although this is sufficient as a proof of concept, it would be interesting to know whether demographic and/or physiological factors influence the performance of patients using this system. Further data are needed. Can it benefit patients of all etiologies? Are there differences between men and women? Are there age-related differences?

In conclusion, our study is one of the first to combine a detector of odorant molecules with a neural stimulator to allow patients with olfactory dysfunction to detect and discriminate different odors. We have shown the preservation of somatosensory trigeminal function in anosmic patients, excellent patient performance in terms of ES detection, and more moderate performance in terms of stimulus discrimination. Our study did not include a training period, and we anticipate that discrimination performance will improve with learning via neural plasticity. Stimulation parameters also require optimization. We believe that individuals will be able to associate the substitute ES with odors. This substitution method, even if it is not yet optimal and does not allow patients to smell real odors, may, therefore, develop into a genuine first technical solution that we could imagine offering to patients. However, if, in future, it becomes advantageous to perform central stimulation of the olfactory brain, then our method may be applied with a simple replacement of the stimulation module.

## MATERIALS AND METHODS

Further information related to participants, materials, and protocols is provided in the Supplementary Materials.

### General information

Experiments were performed in Lyon (France, experiment 1) under ethical approval of the French Sud-Est and Outre-Mer Committee for the Protection of Individuals 4 (IRB: IORG0009855, #21.03.05.82349/CPP2021-03-028a/2021- A00661-40), Thessaloniki (Greece, experiments 2 and 4) with approval by the Decision of the Research Ethics and Deontology Committee of the Aristotle University of Thessaloniki (application number 59718/2021), and Dresden (Germany, experiments 3 and 4) approved by the Ethics Committee at the Universitätsklinikum Gustav Carl Carus at the TU Dresden (application number BO EK 400082021). All volunteers were asked to read an information sheet and sign a consent form relating to the experiment. Once these documents had been read and signed, participants were asked to complete a demographic questionnaire (age, sex, medical history, etc.). The participants’ sense of smell was assessed using the Sniffin Stick identification test (*83*). An assessment was also made of trigeminal function. For experiment 1, trigeminal sensitivity was assessed using a questionnaire and a psychophysical test consisting of the presentation of trigeminal (menthol) and nontrigeminal (vanillin) stimuli. In experiment 2, trigeminal sensitivity was assessed using the AmmoLa test, and, in experiments 3 and 4, the AmmoLa test, a test of sensitivity to CO_2_, and a lateralization test were used (see the Supplementary Materials).

### Experimental design of experiment 1

The experimental procedure included three blocks (see Supplementary Text S3). The first part of this was to establish a baseline as follows: Once the device was installed (Fig. 2A), the detection threshold for ES was measured. The participant was then asked to evaluate the intensity, hedonic valence, and the sensations of warmth and irritation evoked by a stimulus corresponding to the detection threshold and a stimulus of twice the threshold intensity. These responses were given verbally using visual analog scales (VASs): intensity (0 = not detected stimulation at all, 10 = very intense), pleasantness (−5 = very unpleasant, 5 = very pleasant), warmth (0 = not at all warm, 10 = very warm), and irritation (0 = not at all irritating, 10 = very irritating).

Having established the baseline response, the experiment proceeded to an investigation of whether the participants could distinguish between patterns of ES, which could be associated with specific olfactory stimuli (block 2). Here, a sequence of 12 trials was presented, each trial corresponding to a stimulation pattern associated with a class of olfactory stimuli detected by the artificial nose. The patterns were identical in terms of duration of the electrical pulses (500 μs) and their intensity (current set at twice threshold). However, they differed in the number of electrical pulses per pattern and the duration between pulses. Four patterns were set up: pattern #1 (two pulses separated by 400 ms, triggered by the smell of lilac); pattern #2 (no ES, the smell of banana serving as a stimulus for the artificial nose); pattern #3 (four pulses separated by 100 ms, triggered by the smell of raspberry); and pattern #4 (four pulses separated by 400 ms, triggered by the smell of fish). The 12 stimulation pattern trials were presented to the participants in the following pseudorandom sequence: 1-3-2-1-1-4-3-3-2-4-4-1. This sequence made it possible to integrate the three possible pairs comparing control and electrical stimuli (3-2 in duplicate, 2-1, and 2-4), the three possible pairs comparing different electrical stimuli (4-1 in duplicate, 1-3, and 4-3), and the three possible pairs comparing identical electrical stimuli (1-1, 3-3, and 4-4). For the experimental task, after each trial, the participant had to (i) indicate whether the stimulus was identical to or different from the previous stimulus and (ii) evaluate the sensation perceived using the same four-dimensional VAS, as in block 1. Responses were provided verbally.

The final part of our experiment (block 3) was to ask participants to associate each of the real stimulation patterns (#1, #3, and #4) with a category of odors. The stimulations were presented, in turn, and participants were asked to choose between floral, fruity, and rotten fish. Any of the three choices was possible for any of the stimuli regardless of previous choices.

### Experimental design of experiment 2

The experimental procedure included four blocks (see Supplementary Text S4). Block 1 (electrical threshold baseline measure) was the same as that used in experiment 1. Block 2 consisted of a stimulation detection task, which, unlike the block 2 task of experiment 1, was separated from the discrimination task. Participants were presented with a sequence of 12 trials. The same four stimulation patterns used in experiment 1 (patterns #1, #2, #3, and #4) were presented to the participants using the following pseudorandomized sequence: 1-2-3-4-2-2-4-2-3-2-2-1, which includes six dummy stimulations and six real stimulations. After each trial, the participant was asked to assess perceived sensation using the same VAS as those used previously. Block 3 was designed to determine whether the participants could discriminate between different ES patterns. Participants were presented with a sequence of 10 trials including only real electrical patterns (#1, #3, and #4) with an interval between each stimulation pattern of 60 s. The order of presentation of the patterns was 3-4-1-4-4-3-1-1-3-3, which provides three similar pairs (1-1, 3-3, and 4-4) and six different pairs (1-3, 1-4, 3-4, 3-1, 4-1, and 4-3). The participant was asked to indicate verbally whether the stimulus was the same as the previous stimulus in terms of intensity and quality (except for the first stimulus, which was not preceded by any stimulus) on a VAS [0, 10] where 0 was not at all the same and 10 was exactly the same. Last, block 4 was a stimulation/odor category association task identical to the last block of experiment 1.

### Experimental design of experiment 3

The protocol was identical to that of experiment 2, except that only six trials were used in block 2 in the following pseudorandomized sequence: 1-2-3-4-2-2, rather than the 12 trials of experiment 2 (see Supplementary Text S5). The stimulations were generated without the artificial nose. This meant, first, that participants did not receive the auditory preparatory countdown before each stimulation that participants in experiment 2 received and, second, that the time between stimulation patterns was determined by the human experimenter, rather than the e-nose software.

### Experimental design of experiment 4

There were three blocks (see Supplementary Text S6). Block 1 was the baseline measurement identical to those for experiments 1 to 3. Instead of three, experiment 4 used only two ES patterns (#1 and #3). Block 2 was a brief familiarization phase. The two different stimulation patterns were presented in pairs. Each pair was presented twice in the following order: pair 1 (pattern #1 then pattern #3) and then pair 2 (pattern #3 then pattern #1). Within a pair, after a pattern was presented, participants were asked to rate its hedonic valence using a VAS ranging from −5 (very unpleasant) to +5 (very pleasant). After presentation of both patterns in the pair, participants were asked to rank the two patterns from most preferred to least preferred. Block 3 was the discrimination task. The two stimulation patterns were presented in triplets. Each triplet was made up of two identical patterns and one different pattern. Within triplets, stimulation patterns were separated by 10s. After presentation of all three stimulations in a triplet, the participants’ task was to identify the different pattern (intruder). Successive triplets were separated by 60s. Each of the six possible combinations of the two patterns was presented twice in a pseudorandom order. Further details are provided in the Supplementary Materials.

### Statistical analysis

The jamovi software package was used for statistical analysis (*84*, *85*). First, analysis of ES detection threshold data for each individual experiment (experiments 1 to 4) was descriptive given the limited number of individuals within each subgroup (normosmic, hyposmic, and anosmic). However, we also present a meta-analysis of all 65 study participants, comparing the thresholds of normosmic (*n* = 13), hyposmic (*n* = 30), and anosmic (*n* = 22) participants (including the two normosmic participants excluded from analysis of experiments 2 and 3). Levene’s test showed that the variances are not homogeneous (*P* = 0.001), and the Shapiro-Wilk test showed that the distributions are not normal (*P* < 0.001). This analysis was, therefore, carried out using the Kruskal-Wallis test for comparisons of three or more variables with pairwise comparisons made using the Dwass-Steel-Critchlow-Fligner algorithm that adjusts for multiple comparisons.

Second, for data on hedonic perception, intensity, warmth, and irritation, for threshold and double-threshold stimuli, the Shapiro-Wilk test showed that the distributions are not normal and Levene’s test showed that the assumption of equal variances was not obeyed. A Wilcoxon signed-rank test was, therefore, used for comparing threshold scores to double-threshold scores for the perception of intensity, hedonicity, warmth, and irritation, separately. This analysis was carried out in the same way for experiments 1 to 4 and provides the measures of statistical significance illustrated on the figures. In experiment 1, there was only one nonzero rating of heat; in experiment 2, three nonzero ratings; experiment 3, two nonzero ratings; and experiment 4, one nonzero rating. Statistics for this perception are, therefore, not reliable. Data from all four experiments were also combined, and the analysis was performed in the same way in the meta-analysis.

Third, data from the intensity, hedonics, warmth, and irritation ratings in the detection and/or discrimination task were subjected to a Shapiro-Wilk normality test for the distributions in experiments 1 to 3, followed by statistical comparisons between patterns (Kruskal-Wallis test). Practically, in experiment 1, data from all individuals (*n* = 9) were combined with two types of comparisons: To assess participants’ detection abilities, we performed analyses comparing perceived intensity for pattern #2 (control stimulation), with stimulation patterns #1, #3, and #4. Then, to assess perceptual differences between stimulation patterns, analyses comparing patterns #1, #3, and #4 were performed. For experiment 2, the same analyses combining all participants (*n* = 12) were performed, with the addition of separate analyses for the normosmic and individuals with olfactory dysfunction. Similarly, the analysis for experiment 3 was carried out first combining all participants (*n* = 21) and then carrying out separate analyses for hyposmic (*n* = 11) and anosmic (*n* = 9) participants. Last, for experiment 4, patterns #1 and #3 were analyzed by comparing the percentage of individuals who preferred either pattern versus the chance level (50%). Ratings of hedonicity for the two patterns were compared using a Wilcoxon signed-rank test (Levene’s test was significant).

Fourth, discrimination ability was analyzed descriptively for experiment 1, considering the percentage of correct discrimination per participant, and with inferential analyses for the other experiments. In experiment 2, we made comparisons of similarity scores in terms of intensity and quality, between similar versus different pairs. For each participant, scores for similar pairs and scores for different pairs were averaged. A comparison was then made using the Wilcoxon signed-rank test. This analysis was carried out in three ways: on the whole sample (*n* = 12), in normosmic participants only (*n* = 6), and in participants with olfactory dysfunction only (*n* = 6). Experiment 3 used the same statistical tests with the following comparisons: on the whole sample (*n* = 21), in hyposmic participants only (*n* = 11), and in anosmic participants only (*n* = 9). Last, in experiment 4, the percentage of correct discrimination for each participant was calculated. These results were compared to the null hypothesis of a success rate of 33.3% (chance) using the Wilcoxon signed-rank test. All tests are two sided, except otherwise stated in the text.

## Supplementary Materials

### The PDF file includes:

Supplementary Text S1 to S7 Figs. S1 to S4 Table S1 Legend for data S1 References
