# Supplementary material for: Substitution of human olfaction by the trigeminal system
Source: Sci Adv. 2025 Nov 26;11(48):eadu7926. doi: 10.1126/sciadv.adu7926 (PMC12652247; doi:10.1126/sciadv.adu7926)
Supplement: Supplementary file 1 — Supplementary Text S1 to S7 Figs. S1 to S4 Table S1 Legend for data S1 References [file sciadv.adu7926_sm.pdf]

Science Advances
AAAS

Supplementary Materials for

Substitution of human olfaction by the trigeminal system

Halina B. Stanley *et al.*

Corresponding author: Moustafa Bensafi, moustafa.bensafi@cnrs.fr,
Thomas Hummel, thomas.hummel@tu-dresden.de

Sci. Adv. **11**, eadu7926 (2025)
DOI: 10.1126/sciadv.adu7926

The PDF file includes:

Supplementary Text S1 to S7
Figs. S1 to S4
Table S1
Legend for data S1
References

Other Supplementary Material for this manuscript includes the following:

Data S1

SI #1: Methods of detection and stimulation.

We use an optical smell sensor (Neose-Advance® device from Aryballe Technologie) (**Sup Fig 1A**). It includes a pump that draws air into microfluidics tubes and then passes it through an array of bio-functionalized Mach-Zehnder interferometers manufactured on a silicon substrate. The optical interference pattern produced can be used as a “fingerprint” for the odor or mixture of odors (VOC). The response time is around 1 second, but several seconds are needed for the VOC to desorb from the biochemical sensors before another olfactory stimulus can be detected reliably. A wide variety of VOC can be detected by this device.

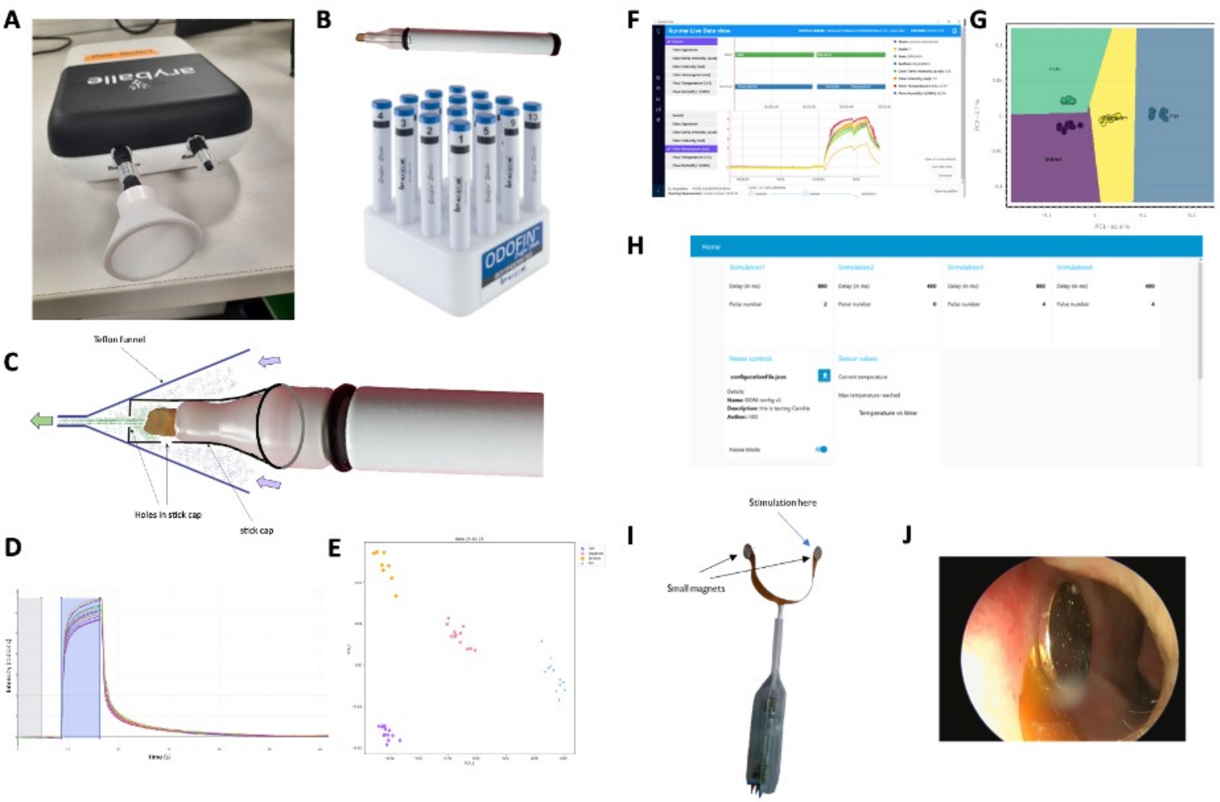

Sup Figure 1. Sensor and stimulation devices. A. The VOC detector (Neose-Advance®) fitted with a Teflon funnel. B. Commercially available “Sniffin Sticks”. C. Connecting the “Sniffin Stick” to the Neose-Advance®. A teflon funnel protects the airflow from the environment. Air is drawn over the felt wick by the pump in the artificial nose, collecting vapour. D. Neose-Advance® signal obtained from a commercially available Sniffin Stick solution (lilac) using the setup illustrated in C (the timeframe of injection is highlighted in blue; a useful signal is obtained within 10s). E. The "fingerprints" of the four odors are distinguishable. F. Aryballe software with real-time VOC classification. G. Classification of Neose-Advance® "fingerprint" data. H. Graphical user interface to drive the stimulation. I. Magnetic "clip" style stimulator. The buttons are small magnets. One branch (only) carries the wires for the stimulation. J. endoscopic image of the stimulator in contact with the anterior septum.

A good discrimination between VOCs requires good signal to noise and a good classification requires reproducible signals. For these experiments we chose to use the Burghart CE Standard test solutions (EN ISO 13485:2016) contained in ODOFIN^{TM} Sniffin’Sticks (**Sup Fig 1B**). These sticks consist of a pen

body which contains a liquid reservoir and a felt tip that wicks the liquid to the air. To adapt this support to the entrance of our detection device, we developed a system where sticks with judiciously pierced caps are presented to a Teflon funnel fixed to the microfluidics input tube (**Sup Fig 1C**). This produces highly reproducible signals, with fast dynamics and no contamination (**Sup Fig 1D**). We identified four odors representing different human perception paradigms that are reliably discriminated by the Neose-Advance® (**Sup Fig 1E**). They are: lilac (pleasant; inedible), banana (pleasant; edible), raspberry (pleasant; edible), rotten fish (unpleasant; inedible) all supplied by www.burghart-mt.de.

Sup Fig 1F depicts the software used to collect the data from the sensors and calculate the odor "fingerprint" of the VOC. A Python script enables classification the VOC from the PCA analysis of the odor "fingerprints" in real-time (based on previously collected calibration data) (**Sup Fig 1G**) and a graphical user interface (node-red code) maps the VOC class to a stimulation pattern (**Sup Fig 1H**).

Based on previous studies (*86*), we choose to stimulate at the anterior septum. This is easy to access and allows us to use a simple magnetic clip-style stimulator which maintains good contact between the electrodes and the nasal mucosa (*87*) (**Sup Fig 1I, Sup Fig 1J**).

SI #2. Experimental protocol for fitting intranasal stimulator & running a sequence of stimulations.

The stimulator (magnetic clip type) at the level of the anterior septum, was positioned on the anterior septum 1 cm from the vestibule and 2 cm above the base of the nasal cavity using a pair of non-magnetic tweezers and an endoscope (**Sup Fig 2A-B**). Once the electrode was in contact with the mucosa, the stimulator was held in place by an external support which consisted of a pair of glasses (Experiments 1 & 3) or a nose protector mask (Experiments 2).

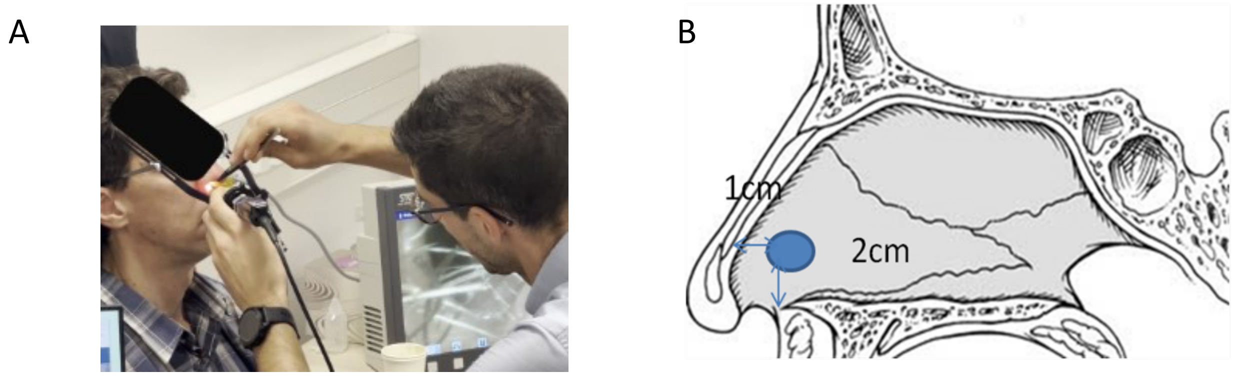

Sup Figure 2. Experimental protocol (example for Experiment 1). A. Installation of the magnetic clip style stimulator by the ENT doctor under endoscopy. B. Schematic illustrating stimulation position on the nasal septum

The electrical stimulation pulses produced by a Digitimer DS7A were controlled by a computer via a customized electronic board with a microcontroller. The Digitimer was set to produce pulses of 500µs duration, maximum 250V. Current was set manually based on individual threshold and then fixed for the duration of the experiment. (**Sup Fig 3A-B**). The mapping between the odor detected by the e-nose and the sequence of stimulations (number of stimulations & delay between stimulations) was coded prior to the start of the experiment.

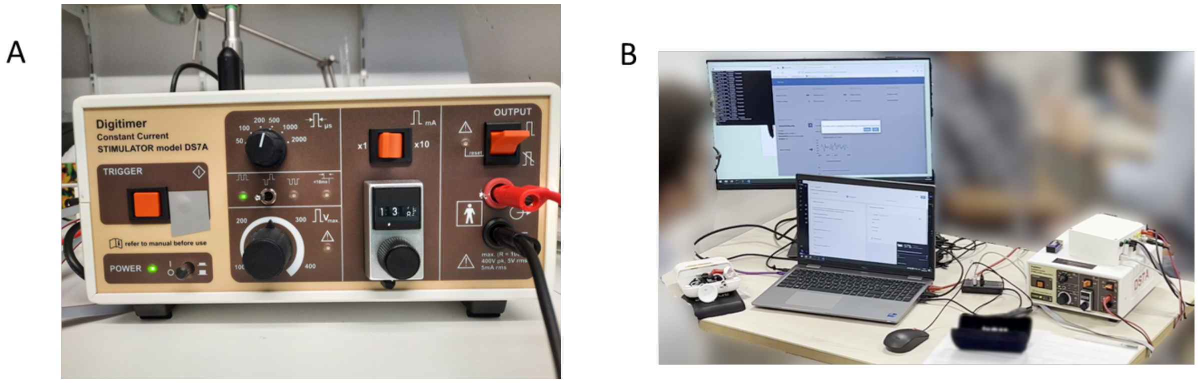

Sup Figure 3. Running sequence of stimulations A. Digitimer DS7A settings. B. Digitimer with custom electronics, computer control and e-nose.

The experiment began with an auditory warning (steam train arriving whistle) followed by three tones of increasing pitch. The experimenter then presented one of the four odors contained in Sniffin Sticks to the e-nose. The e-nose detected the odorant and its software classified it into one of the four pre-programmed

possibilities. Ten seconds after the rising tones the experimenter & the subject heard a sequence of 3 tones with decreasing pitch. This signaled to the experimenter to remove the odorant stimulation from the e-nose which began its desorption, cleaning, phase in readiness for the next odor. These tones were also the signal to the subject to expect to receive a stimulation sequence. The software sent the appropriate sequence of triggers to the digitimer automatically. There was then a pause of 60s during which oral feedback was provided by the subject, before the whole sequence was repeated, beginning with the warning noise of the steam train whistle.

SI #3. Detailed methods for Experiment 1.

Overall presentation. The experiment consisted of 3 block sessions. First, the detection threshold for electrical stimulation was defined for each participant (Block 1). Once the threshold had been measured, a series of patterns of stimulation (with a current fixed at double the participant's measured threshold) was sent to the participant (Blocks 2 and 3). Here, each molecule was classified by the artificial nose and mapped to a specific stimulation pattern so as to deliver a unique electrical impulse for each odorant molecule. Several trials were performed, and for each of these trials, participants had to determine whether electrical stimulation was present (we included a dummy control case where the stimulator sent zero electrical stimulation) and whether this stimulation was different from that previously perceived (discrimination task). In addition, participants were asked to describe each electrical stimulation according to different perceptual dimensions (intensity, hedonic character, etc.). The definition of the association between a given odorant molecule and the stimulation pattern was arbitrary for this first experiment, however, the data collected in this first experiment helped us to characterize these stimulation patterns in terms of perception and pleasantness for the subsequent experiments.

Participants. A total of 9 volunteers (5 normosmics; 4 patients with olfactory disorder from 4 different etiologies: post-viral, post-traumatic, sinonasal, congenital; 6 men, 3 women, age 43.4 (±5.2) years, range [23,63] years) were included in the study. The participants' sense of smell was assessed using the Sniffin Stick identification test *(83)*. In addition, their trigeminal sensitivity was assessed using a questionnaire and a psychophysical test consisting of the presentation of trigeminal (e.g. menthol) and non-trigeminal (e.g. vanillin) stimuli. The test of olfactory function confirmed that the five volunteers without known olfactory impairment did, indeed, have normal olfaction (Sniffin stick scores in the range [13,16]) and that three of the four patients were anosmic (scores in the range [1,4]). However, the post COVID-19 patient was found to have recovered some olfactory function (classified as hyposmic, average score 11). All demonstrated more trigeminal responses to menthol (trigeminal stimulus) compared to vanilla (known as pure olfactory stimulus).

Installation. The experimental sessions were carried out in an air-conditioned room, where the temperature was maintained between 21°C and 22°C. The device comprising the artificial nose, the odor stimuli, the stimulator and the computer (linking the artificial nose and the stimulator), was positioned on a table with the two experimenters on one side (one managing the device and the other recording the participant's responses) and the participant on the other side of the table, comfortably seated in a chair with his head in a stable position. An ENT doctor, seated next to the participant, was present throughout the experimental session. Before starting the protocol, the ENT doctor installed (under endoscopy) the stimulator (magnetic clip type) at the level of the anterior septum, 1 cm from the vestibule and 2 cm above the base of the nasal cavity. Nasal endoscopy monitoring ensured that the stimulation electrode was in contact with the respiratory mucosa. Once the electrode was in contact with the mucosa, the stimulator was held in place by an external support. It should be noted that the stimulator stimulated only one side of the nasal cavity, so the stimulation side - left vs. right nostril - was counterbalanced across participants.

Protocol. The experimental procedure included 3 blocks. In **Block 1**, the aim was to measure the detection threshold to electrical stimulation sent to the nasal cavity. The instruction given to the participants was as follows: *"We are going to send a series of bioelectrical stimulations into your nasal cavity. These stimulations can range from nothing, to extremely low intensity (and virtually undetectable), to detectable electrical stimulation. Each trial begins with a countdown from the experimenter: "3, 2 then 1". After "1", the stimulation is sent and you must decide whether or not you detected an electrical impulse by answering verbally: "yes" or "no"".* During threshold determination, the interval between

stimuli was around 10 seconds (this interval was not fixed and depended on the participant's speed of response). The current was increased from 0.00 mA in steps of 0.10 mA until the participant felt the electrical stimulation twice in a row. The current intensity was then decreased by steps of 0.05 mA until the individual could no longer detect the stimulation. These turning points were repeated seven times (with steps 0.05 mA) and the threshold was calculated by averaging the last four.
After determining the detection threshold, the experimenter presented the participant with two electrical stimuli: a stimulus corresponding to the detection threshold, and a stimulus with twice the intensity of the detection threshold. For each of these two stimuli, the participant was asked to evaluate them according to different perceptual criteria using a visual analogue scale: intensity [0=not at all intense,10=very intense], pleasantness [-5=very unpleasant,5=very pleasant], warmth [0=not at all warm,10=very warm] & irritation [0=not at all irritating,10=very irritating]. Responses were provided verbally.

The aim of **Block 2** was to determine whether the participants detected and discriminated different electrical stimulation patterns. Participants were presented with a sequence of 12 trials. Each trial corresponded to a stimulation pattern, which was associated with an odor class determined by the artificial olfactory sensors. For each stimulation pattern, the duration of the electrical pulses (500µs) and their intensity (current fixed at double the participant's measured threshold) were constant. The patterns differed in two parameters: the number of electrical pulses per pattern and the time between pulses. Pattern characteristics were as follows: pattern #1 (2 pulses separated by 400ms, triggered by the lilac odor); pattern #2 (no electrical stimulation, banana odor serving as the stimulus for the artificial nose); pattern #3 (4 pulses separated by 100ms, triggered by the raspberry odor); pattern #4 (4 pulses separated by 400ms, triggered by the fish odor). A total of 12 trials of patterns of stimulation were presented to the participants using the following pseudorandomized sequence: 1-3-2-1-1-4-3-3-2-4-4-1. This sequence thus made it possible to integrate the 3 possible pairs comparing control and electrical stimuli (3-2 in duplicate, 2-1, 2-4), the 3 possible pairs comparing different electrical stimuli (4-1 in duplicate, 1-3, 4-3) and the 3 possible pairs comparing identical electrical stimuli (1-1, 3-3, 4-4). We deliberately kept the sequence of stimulations short in order to minimize possible effects of fatigue. The time between two stimulation patterns was fixed at 60 seconds. During this time the participant was asked to perform two tasks: i/ indicate whether the stimulus was the same as or different from the previous stimulus (except for the first stimulus, which was not preceded by any stimulus), and ii/ assess perceived sensation using 4-dimensional visual analogue scales, as in block 1: intensity [0=not at all intense,10=very intense], pleasantness [-5=very unpleasant,5=very pleasant], warmth [0=not at all warmth,10=very warmth] & irritation [0=not at all irritation,10=very irritating]. Responses were provided verbally. Specifically, participants were given the following instructions. *"In this block, we are going to stimulate your nasal cavity with detectable bioelectric stimulation of the same nature as those you experienced during the first block. Each trial begins with a warning sound of a "train whistle", followed by tones of increasing pitch. These auditory signals will be followed by tones of decreasing pitch, during which electrical stimulation will be sent into your nasal cavity. It's during this period that you should assess i/ whether the stimulus was the same as or different from the previous stimulus (except for the first stimulus, which was not preceded by any stimulus), and ii/ assess perceived sensation using 4-dimensional visual analogue scales for intensity [0=not at all intense,10=very intense], pleasantness [-5=very unpleasant,5=very pleasant], warmth [0=not at all warmth,10=very warmth] and irritation [0=not at all irritation,10=very irritating].".*

The aim of **Block 3** was to evaluate the concordance between the stimulation pattern (#1, #3 or #4) and the olfactory class (lilac, raspberry, fish) according to the participants. In other words, we were investigating how much inherent sense these mappings made to participants. To do this, we presented each stimulation pattern (except the control pattern #2) to the participants and asked them to choose one of 3 possible categories with which to associate the ES: flower, fruit, and rotten fish. Participants could choose any of the three categories for any of the three stimulation patterns regardless of previous choices. Instructions given to the participants were: *"In this block, we will stimulate your nasal cavity with 3 detectable bioelectric stimuli of the same nature as those you experienced in block 2. Each trial begins with the countdown from the computer. Your task will be to associate each of these stimuli with a*

category of odors, which may be floral, fruity or rotten fish. You can choose the same odor category for different stimuli, but you can also choose different categories for different bio-electrical stimuli. Any combination is possible.".

SI #4. Detailed methods for Experiment 2.

Overall presentation. Experiment 2 was performed in Thessaloniki (Greece). The protocol used was very similar to that used in Experiment 1, however the discrimination task was separated from the detection task in order to reduce cognitive effort.

Participants. A total of 12 volunteers (6 normosmics and 6 patients with olfactory disorder from various etiologies – 1 post-traumatic, 3 post-viral and 2 with sino-nasal disease, 2 male and 10 female, age 45.3 (±3.9) years, range [27,69] years) were included in the study approved by the Decision of the Research Ethics and Deontology Committee of the Aristotle University of Thessaloniki (application number 59718/2021). All volunteers were asked to read an information sheet and sign a consent form relating to the experiment. Once these documents had been read and signed, participants were asked to complete a demographic questionnaire as in Experiment 1. The participants' sense of smell was assessed using the Sniffin Stick identification test *(83).* Among patients, 5 were hyposmics and one was anosmic (Sniffin' Stick score 11). In addition, their trigeminal sensitivity was assessed using the AmmoLa test. The AmmoLa test is a commercially available lipstick-like container that contains traces of lavender perceptually dominated by a strong irritation produced by ammonium. The ratings of its irritation after sniffing on a scale from 0 (no perception) to 100 (extremely irritating) give an idea of the trigeminal sensitivity. The AmmoLa scores were between 50 and 80 for all patients. For the healthy subjects, Sniffin' Stick mean score was 33.8, with a range of [31, 37], and AmmoLa test scores ranged from 80 to 100.

Installation. The set-up was identical to that used in Experiment 1, the only difference being that that for ergonomics and comfort reasons, the stimulator clip was stabilized with a new support (**Fig 3A**, main paper).

Protocol. The experimental procedure included 4 blocks. **Block 1** (electrical threshold baseline measure) was the same as that used in Experiment 1. The aim of the **Block 2** was to determine whether the participants detected different electrical stimulation patterns compared to a control stimulation (zero stimulation intensity). To this end, participants were presented with a sequence of 12 trials. The same 4 stimulation patterns used in Experiment 1 (patterns #1, #2, #3, #4) were used to construct the sequence that included a total of 6 trials of pattern #2 (no electrical stimulation) and 6 trials of patterns #1, 2 and 4 (6 in total, 2 per pattern). These stimulations were presented to the participants using the following pseudorandomized sequence: 1-2-3-4-2-2-4-2-3-2-2-1. Here, the time separating the presentation of two stimulation patterns was 60 seconds. As in Experiment 1, after each trial, the participant was asked to assess perceived sensation using 4-dimensional visual analogue scales, as in block 1: intensity [0=not detected stimulation at all,10=very intense], pleasantness [-5=very unpleasant,5=very pleasant], warmth [0=not at all warm,10=very warm] & irritation [0=not at all irritating,10=very irritating]. Responses were provided verbally. Specifically, participants were given the following instructions. *"As in the first block, we are going to stimulate your nasal cavity with bioelectric stimulation of varying intensity, ranging from nothing, to extremely low intensity (and virtually undetectable), to detectable electrical stimulation. The stimuli will be of the same nature as those you experienced during the first block. Each trial begins with a warning sound of a "train" (during which time the experimenter will place a scented stick on the artificial nose), followed by tones of increasing pitch. These auditory signals will be followed by tones of decreasing pitch, during which electrical stimulation will be sent into your nasal cavity. It's during this period that you should assess whether or not you are feeling the stimulation. Specifically, your task will be to indicate whether you detect electric stimulation using an intensity scale ranging from 0 (no stimulation) to 10 (very intense stimulation). If you detect any stimulation, you will be asked to rate it according to the following criteria: pleasantness [-5=very unpleasant,5=very pleasant], warmth [0=not at all warm,10=very warm] & irritation [0=not at all irritating,10=very irritating]."*.

The aim **Block 3** was to determine whether the participants could discriminate between different electrical stimulations. To this end, participants were presented with a sequence of 10 trials including only electrical patterns (#1, #3 and #4) with an interval between stimulation of 60 seconds. The order of presentation of the patterns was as follows: 3-4-1-4-4-3-1-1-3-3. With such a stimulation sequence, we therefore have 3 similar pairs (1-1, 3-3 and 4-4), 3 different pairs (1-3, 1-4 and 3-4) and 3 other different pairs but in reverse order (3-1, 4-1 and 4-3). As in block 2, each trial began with a countdown by the computer after which the participant was asked to indicate whether the stimulus was the same as, or different from, the previous stimulus in terms of intensity and quality (except for the first stimulus, which was not preceded by any stimulus). Responses were provided verbally. Specifically, participants were given the following instructions. *"In this block, we are going to stimulate your nasal cavity with detectable bioelectric stimulation of the same nature as those you experienced during the first and the second blocks. Each trial begins with a warning sound of a "train", followed by tones of increasing pitch. These auditory signals will be followed by tones of decreasing pitch, during which electrical stimulation will be sent into your nasal cavity. It's during this period that you should assess whether the stimulus was the same as, or different in terms of both intensity and quality from, the previous stimulus (except for the first stimulus, which was not preceded by any stimulus). To this end, you will provide your answers using visual analogue scales: intensity (0: not at all the same in intensity; 10: exactly the same in intensity"), quality (0: not at all the same in quality; 10: exactly the same in quality")."*.

Finally, **Block 4** consisted in the stimulation/odor association task was identical to that used in Experiment 1.

SI #5. Detailed methods for Experiment 3.

Overall presentation. Experiment 3 was performed in Dresden (Germany). Here the aim was to replicate Experiment 2 in a larger cohort of patients, but for simplicity we used only the stimulator and not the e-nose. As a result, the participants received the stimuli without an auditory pre-stimulus countdown because this function was managed by the e-nose. Lastly, to lighten the experiment, whereas in Thessaloniki, the detection task (Block 2) involved 12 trials, it involved only 6 in Dresden. Apart from these differences, the protocol was the same as in Experiment 2.

Participants. A total of 21 volunteers (9 anosmic, 11 hyposmic and 1 normosmic; 16 post-viral, 3 sinonasal, 1 idiopathic and 1 post-surgical; 6 male and 15 female; age 59.3 (±1.8) years, range [38,76] years) were included in the study approved by the Ethics Committee at the Universitätsklinikum Gustav Carl Carus at the TU Dresden (application number BO EK 400082021). One participant had a score of 31.75 (normosmic) and was excluded from analysis of this experiment, although included in meta-analyses. As in Experiments 1 and 2, all volunteers were asked to read an information sheet and sign a consent form relating to the experiment. Once these documents had been read and signed, participants were asked to complete a demographic questionnaire as in Experiments 1 and 2. The patients' sense of smell was assessed using the Sniffin Stick identification test *(83)*. The 20 patients had scores in the range [9, 29.5] and nine of them patients were anosmic (scores in the range [9, 13.5]). In addition, their trigeminal sensitivity was assessed using three tests. The first one is the AmmoLa (as in Experiment 2). The second test was a portable device releasing short puffs of CO_2 into nasal cannulas. The intensity of the puffs (modelled by the duration of the pulses) varies in a staircase procedure similar to the threshold test from the Sniffin' Sticks, with seven turning points. A threshold is then calculated with the mean of the last four turning points. Lastly, a lateralization test was performed with a trigeminal eucalyptus smell. In this task, two identical airflows were applied to both nostrils using a handheld "squeezing device" which releases the same amount of air simultaneously to the left and right nostrils. One side received the target odorant, while the other side received odorless air. The sides of the odorant stimulation were changed in pseudo-randomized order. If the odorant has a trigeminal component, and if the patient has a good trigeminal sensitivity, the success rate in detecting the stimulated nostril increases significantly (*88*). All demonstrated sufficient trigeminal responses with at least one of the three trigeminal tests (in general, patients had high-intensity ratings to trigeminal stimuli (i.e., "AmmoLa scores") between 90 and 100, or CO_2 threshold lower than 1505msor lateralization scores higher than 10) except for one patient who was not tested for trigeminal sensitivity.

Installation. The set-up was identical to that used in Experiment 1, except that the e-nose detection was omitted and the stimulation sequence was triggered directly by the experimenter. The stimulation device support from Experiment 1 was used (**Fig 4A**, main paper).

Protocol. The experimental procedure included 4 blocks. **Block 1** (electrical threshold measure) was the same as that used in Experiments 1 and 2. **Block 2** was the same as that used in Experiment 2 except that only 6 trials were used in the following pseudorandomized sequence: 1-2-3-4-2-2. For **Block 3**, we used the exact same sequence as that of Experiment 2 for subjects 1 to 15 and sequence 3-3-1-1-3-4-1-4-3-4 for subjects 16-21. (***Note:*** *If data are restricted to subjects 1 to 15 (to compare with Experiment 2) participants could not discriminate between identical and different pairs (W= 74, p=0.460) by intensity and (W=64, p=0.490) by quality).* However, because we only used the stimulator part of the device, the signal preceding and during each trial (warning sound of a "train" followed by tones of increasing/decreasing pitch) was not presented for either Block 2 or 3. Finally, for Block 4, the stimulation/odor association task was identical to that used in Experiments 1 and 2.

SI #6. Detailed methods for Experiment 4.

Overall presentation. Experiment 4 was performed in both Dresden (Germany) and Thessaloniki (Greece). In Thessaloniki the nose protector visor stimulator support was used (**Fig 3A**, main paper) and in Dresden the glasses support (**Fig 4A**, main paper). In all other respects the protocols and materials were identical in the two sites. This experiment was aimed at testing participants' discrimination ability. In light of the results of Experiments 2 & 3, we included a brief stimulation pattern familiarization sequence, auditory warnings prior to stimulation and a simplified discrimination task.

Participants. A total of 23 volunteers were included in the study (9 anosmic, 13 hyposmic & 1 normosmic, all post-viral, 6 male and 17 female, age 52.0 (±3.0) years, range [19,79] years). One participant had normosmia and was excluded from analysis of this experiment, although included in meta-analyses. All 22 patients were asked to read an information sheet and sign a consent form relating to the experiment, which was approved by the respective ethics committees quoted for Experiments 2 & 3. Once these documents had been read and signed, participants were asked to complete a demographic questionnaire (age, sex, medical history, etc.). The participants' sense of smell was assessed using the Sniffin Stick identification test *(83)*. Their trigeminal sensitivity was assessed using the AmmoLa and lateralization tests (Thessaloniki and Dresden) and CO_2 test (Dresden). The 13 hyposmic subjects had TDI in the range [16.5,27] and the 9 anosmic subjects range [2,14]. There was no correlation between TDI and trigeminal lateralization scores (Pearson r=0.02 [-0.40,0.43]).

Installation. The set-up was identical to that used in Experiment 3. The e-nose detector was omitted, but the entire sequence of stimulation patterns was pre-programmed, including auditory pre-stimulation warnings.

Protocol The experimental procedure included 3 blocks. **Block 1** (electrical threshold measure) was the same as that used in Experiments 1, 2 and 3. **Block 2**, was a brief familiarization of the 2 patterns of electrical stimulation the participants were to receive in block 3 (the discrimination task). The two patterns we used were pattern #1 and pattern #3 as described in Experiment 1 above. They were chosen on the basis of a meta-analysis of the perceptual responses collected in Experiments 1, 2 & 3 (**SI #1)**, in which these patterns were found be perceived to have the greatest difference in intensity of the three real ES (**SI #1)**. These patterns were presented in pairs, and each pair was presented twice in the following order: pair 1 (pattern #1 then pattern #3) then pair 2 (pattern #3 then pattern #1). Within a pair, after a pattern was presented, participants were asked to rate its hedonic valence using a visual scale ranging from -5 (very unpleasant) to +5 (very pleasant). After the presentation of both patterns in the pair, participants were asked to rank the two patterns from most preferred to least preferred. Within a pair, the time separating the presentation of two stimulation patterns was 20 seconds. The time between pairs was 60 seconds. The instructions given to the subjects were as follows: *"We are going to send you **2 different** patterns of electrical stimulation. These patterns will be presented by pair. Within a pair, after the presentation of a given pattern, you will have to perceive it and rate its pleasantness. To do this, you will respond using a visual analogue scale from -5 for very unpleasant, to +5 for very pleasant. Once the two patterns are presented, you will have to rank them from most preferred to least preferred. A total of 2 pairs of patterns will be presented. Note that in the next block of the experiment (block 3), you will have to discriminate these 2 electrical patterns. We therefore ask you to pay particular attention to each of these electrical patterns. Indeed, whereas one of the patterns will consist of 2 small electrical stimuli separated by 400ms each, the other pattern will consist of 4 small electrical stimuli separated by 100ms each."*.

The aim of the **third block** was to determine whether the participants could discriminate between different electrical stimulations. To this end, the stimulation patterns were presented in triplets. Each

triplet was made up of two identical patterns and one different pattern. The time between ES patterns *within* a triplet was 10s and the interval between triplets was 60 seconds. The participants' task was to identify the different pattern (intruder) among the 3 presented in the triplet. With the 2 patterns, it was possible to create 6 triplets: Triplet 1 (pattern #1-#1-#3), Triplet 2 (1-3-1), Triplet 3 (3-1-1), Triplet 4 (3-3-1), Triplet 5 (3-1-3), Triplet 6 (1-3-3). Each of these triplets was presented twice to the participants in the following order: **Triplet 1 – Triplet 3 – Triplet 6 – Triplet 5 – Triplet 2 – Triplet 4 – Triplet 3 – Triplet 5 – Triplet 1 – Triplet 2 – Triplet 6 – Triplet 4.** Due to a technical issue, for 11 participants the final triplet was omitted. Within a triplet, each trial began with a countdown by the computer followed by the stimulation pattern. After the third stimulation pattern, the participant was asked to indicate which of the 3 trials was different within the triplet. Responses were provided verbally and coded by the experimenter.

Specifically, participants were given the following instructions. *"In this block, we will stimulate your nasal cavity with detectable bioelectric stimuli identical to those you experienced in the previous block. The stimuli are presented in triplets. Each triplet is made up of two identical stimulation patterns and one different stimulation pattern. Each triplet trial begins with an audible "train whistle" warning. Then before each ES you will hear tones of increasing frequency followed by tones of decreasing frequency, after which the electrical stimulation pattern is sent into your nasal cavity. For the first and second stimulation patterns of the triplet, you don't need to do anything. But after the third stimulation pattern, you will have to decide which of the 3 trials is different from the other two. You will give your answer verbally, indicating whether it is the first, second or third.".*

SI #7: Associations between ES patterns and olfactory perceptual qualities

Meta-analysis combining data from Experiments 1, 2 and 3 and comparing the different stimulation patterns in terms of perception (hedonic, intensity, warmth and irritation) and their potential association with olfactory qualities.

This analysis examined in a **first step** how the different patterns of electrical stimulation differed in terms of perceptual evaluation (hedonic valence, intensity, warmth and irritation). These perceptual ratings were asked of all participants in the 3 experiments during the detection and/or discrimination tasks. In addition, at the end of all the experimental protocols, the participants were asked to associate each electrical stimulation pattern with one of 3 possible olfactory categories: flower, fruit and rotten fish. For each pattern, they could choose any of the three. Thus, in a **second step**, the present analysis combined the data from the 3 experiments to gain a better understanding of which olfactory class was associated with each stimulation pattern.

Statistical calculations for warmth are not reliable as this perception was essentially absent (zero at 75th percentile). For all other perceptions there were significant differences between the real stimulations (patterns #1, #3 & #4) and the dummy (pattern #2), however, once corrected for multiple comparisons there were no statistically significant differences at the 5% level between the real stimulation patterns (**Sup Fig 4A-D)**.

Nevertheless pattern #3 may be more intense than pattern #1 (p=0.054) (**Sup Table 1** & **Sup Fig 4A**). Both Pattern #3 (p=0.075) & Pattern #4 (p=0.066) may also be less pleasant than pattern #1 (**Sup Fig 4B**). No significant difference appeared between patterns #3 and #4.

Second, for the stimulation pattern/olfactory quality association task, the "rotten fish" quality was chosen much more often for patterns #3 and #4 than for pattern #1 ($\chi^2(2)=18.2$ $p<0.001$) (**Sup Fig 4E**). In contrast, the "floral" quality was chosen more often for pattern #1 than for patterns #3 and #4 ($\chi^2(2)=5.87$ $p=0.053$) (**Sup Fig 4E**). There were no significant differences between choices for "fruity" ($\chi^2(2)=1.00$ $p=0.607$) (**Sup Fig 4E**). The differences between the choices for stimulation #3 and #4 were not statistically different, and for these stimulations all three choices were equally likely.

When these two meta-analyses are **combined**, they suggest that pattern #1 is less intense and less unpleasant than patterns #3 & #4 and people associated it with floral, even fruity qualities rather than rotten fish. There was little in the way of difference between Pattern #3 & Pattern #4; both seem to be more unpleasant and intense than pattern #1, and are more often associated with the rotten fish choice with unpleasant olfactory connotations.

Dwass-Steel-Critchlow-Fligner pairwise comparisons

Pairwise comparisons - Intensity [0,10]

		W	p
1	3	3.274	0.05378
1	4	2.472	0.18761
3	4	-0.719	0.86742

Pairwise comparisons - Hedonicity [-5,5]

		W	p
1	3	-3.0783	0.0753
1	4	-3.1537	0.06625
3	4	-0.0809	0.99822

Pairwise comparisons - Irritation [0,10]

		W	p
1	3	1.687	0.4576
1	4	1.331	0.61441
3	4	-0.271	0.98003

Sup Table 1. Statistical comparisons of the perception of the stimulation patterns. Pair-wise statistical analysis of perceptual differences between stimulation patterns #1, #3 & #4 using the Dwass-Steel-Critchlow-Fligner algorithm

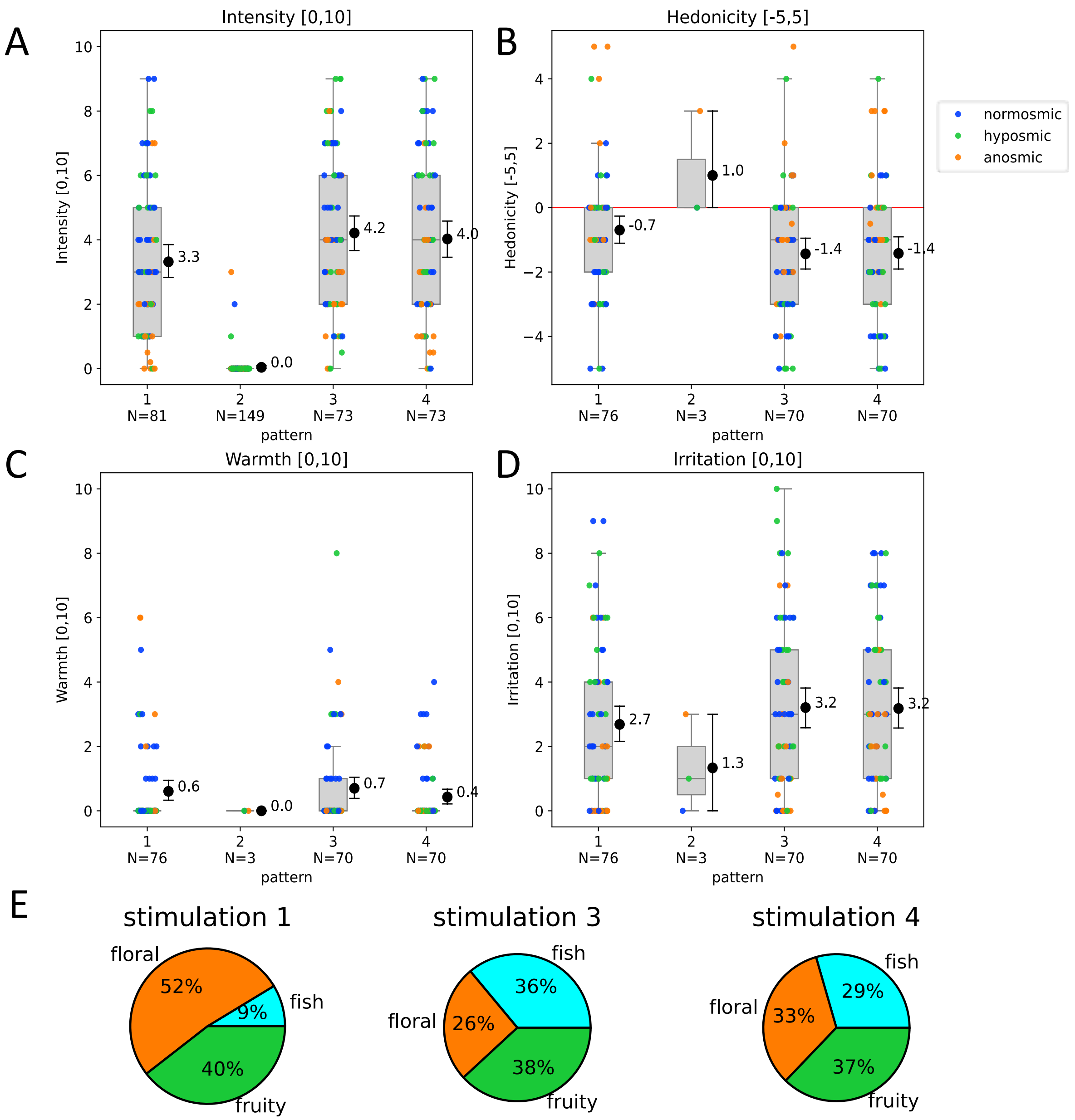

Sup Figure 4. Perceptual and semantic evaluations of the stimulation patterns. Participant's subjective perception of intensity (A), hedonicity (B), warmth (C) and irritation (D) for the 4 different stimulation patterns. Data for hedonicity, warmth & irritation excludes any responses for which the perception of intensity was zero (nothing was felt by the subject). E. percentage of subjects choosing each quality for each stimulation pattern (forced choice). This meta-analysis combines data from Experiments 1, 2 and 3, and includes a total of 42 participants (Note that a normosmic participant, excluded from experiment 3, was included in this meta-analysis).

Caption for the supplementary data.

Supplementary data. These data accompany article « Substitution of human olfaction by the trigeminal system by Stanley, Lipp, Mignot, Weise, Garefis et al. ». Tab description is as follows: General (Overview of subject characteristics by experiment), Exp1-4 Threshold (Threshold for detection of electrical stimulation by subject and experiment), Exp1-4 Stimulation Perception (Perceptual evaluation of electrical stimulation by subject & experiment at threshold and 2x threshold current), Exp1-3 Pattern Perception (Perceptual evaluation of different electrical stimulation patterns by each presentation in the sequence, subject and experiment), Exp1 Pattern discrimination (Sequence of stimulation patterns with subject response "same/different" to previous), Exp2-3 Block3 discrimination (raw discrimination data for experiments 2 & 3), Exp2-3 Pattern discrimination (sorted discrimination data for experiments 2 & 3), Exp4 Preference (preference data comparing patter #1 & pattern #3), Exp4 Pattern discrimination (discrimination data for experiment 4), Exp1-3 cross-modal (Subjects' associations of different concepts to the different stimulation patterns).

REFERENCES AND NOTES

1. R. J. Stevenson, An initial evaluation of the functions of human olfaction. *Chem. Senses* **35**, 3–20 (2010).

2. I. Croy, S. Nordin, T. Hummel, Olfactory disorders and quality of life—An updated review. *Chem. Senses* **39**, 185–194 (2014).

3. T. Hummel, K. L. Whitcroft, P. Andrews, A. Altundag, C. Cinghi, R. M. Costanzo, M. Damm, J. Frasnelli, H. Gudziol, N. Gupta, A. Haehner, E. Holbrook, S. C. Hong, D. Hornung, K. B. Hüttenbrink, R. Kamel, M. Kobayashi, I. Konstantinidis, B. N. Landis, D. A. Leopold, A. Macchi, T. Miwa, R. Moesges, J. Mullol, C. A. Mueller, G. Ottaviano, G. C. Passali, C. Philpott, J. M. Pinto, V. J. Ramakrishnan, P. Rombaux, Y. Roth, R. A. Schlosser, B. Shu, G. Soler, P. Stjärne, B. A. Stuck, J. Vodicka, A. Welge-Luessen, Position paper on olfactory dysfunction. *Rhinol. Suppl.* **54**, 1–30 (2017).

4. C. Manesse, C. Ferdenzi, M. Sabri, M. Bessy, C. Rouby, F. Faure, D. Bellil, S. Jomain, B. N. Landis, M. Hugentobler, M. Cuevas, T. Hummel, M. Bensafi, Dysosmia-associated changes in eating behavior. *Chemosens. Percept.* **10**, 104–113 (2017).

5. C. Ferdenzi, C. Bousquet, P.-E. Aguera, M. Dantec, C. Daudé, L. Fornoni, A. Fournel, A. Kassan, M. Mantel, M. Moranges, E. Moussy, S. Richard Ortegón, C. Rouby, M. Bensafi, Recovery from COVID-19-related olfactory disorders and quality of life: Insights from an observational online study. *Chem. Senses* **46**, bjab028 (2021).

6. C. Manesse, C. Ferdenzi, M. Mantel, M. Sabri, M. Bessy, A. Fournel, F. Faure, D. Bellil, B. M. Landis, M. Hugentobler, A. Giboreau, C. Rouby, M. Bensafi, The prevalence of olfactory deficits and their effects on eating behavior from childhood to old age: A large-scale study in the French population. *Food Qual. Prefer.* **93**, 104273 (2021).

7. A. Brämerson, L. Johansson, L. Ek, S. Nordin, M. Bende, Prevalence of olfactory dysfunction: The Skövde population-based study. *Laryngoscope* **114**, 733–737 (2004).

8. B. N. Landis, C. G. Konnerth, T. Hummel, A study on the frequency of olfactory dysfunction. *Laryngoscope* **114**, 1764–1769 (2004).

9. K. L. Whitcroft, A. Altundag, P. Balungwe, P. Boscolo-Rizzo, R. Douglas, M. L. B. Enecilla, A. W. Fjaeldstad, M. A. Fornazieri, J. Frasnelli, S. Gane, H. Gudziol, N. Gupta, A. Haehner, A. K. Hernandez, E. H. Holbrook, C. Hopkins, J. W. Hsieh, C. Huart, S. Husain, R. Kamel, J. K. Kim, M. Kobayashi, I. Konstantinidis, B. N. Landis, M. Lechner, A. Macchi, P. P. Mazal, I. Miri, T. Miwa, E. Mori, J. Mullol, C. A. Mueller, G. Ottaviano, Z. M. Patel, C. Philpott, J. M. Pinto, V. R. Ramakrishnan, Y. Roth, R. J. Schlosser, P. Stjärne, L. Van Gerven, J. Vodicka, A. Welge-Luessen, P. J. Wormald, T. Hummel, Position paper on olfactory dysfunction: 2023. *Rhinology* **61**, 1–108 (2023).

10. V. Parma, K. Ohla, M. G. Veldhuizen, M. Y. Niv, C. E. Kelly, A. J. Bakke, K. W. Cooper, C. Bouysset, N. Pirastu, M. Dibattista, R. Kaur, M. T. Liuzza, M. Y. Pepino, V. Schöpf, V. Pereda-Loth, S. B. Olsson, R. C. Gerkin, P. R. Domínguez, J. Albayay, M. C. Farruggia, S. Bhutani, A. W. Fjaeldstad, R. Kumar, A. Menini, M. Bensafi, M. Sandell, I. Konstantinidis, A. Di Pizio, F. Genovese, L. Öztürk, T. Thomas-Danguin, J. Frasnelli, S. Boesveldt, Ö. Saatci, L. R. Saraiva, C. Lin, J. Golebiowski, L.-D. Hwang, M. H. Ozdener, M. D. Guàrdia, C. Laudamiel, M. Ritchie, J. Havlícek, D. Pierron, E. Roura, M. Navarro, A. A. Nolden, J. Lim, K. L. Whitcroft, L. R. Colquitt, C. Ferdenzi, E. V. Brindha, A. Altundag, A. Macchi, A. Nunez-Parra, Z. M. Patel, S. Fiorucci, C. M. Philpott, B. C. Smith, J. N. Lundström, C. Mucignat, J. K. Parker, M. van den Brink, M. Schmuker, F. P. S. Fischmeister, T. Heinbockel, V. D. C. Shields, F. Faraji, E. Santamaría, W. E. A. Fredborg, G. Morini, J. K. Olofsson, M. Jalessi, N. Karni, A. D’Errico, R. Alizadeh, R. Pellegrino, P. Meyer, C. Huart, B. Chen, G. M. Soler, M. K. Alwashahi, A. Welge-Lüssen, J. Freiherr, J. H. B. de Groot, H. Klein, M. Okamoto, P. B. Singh, J. W. Hsieh, GCCR Group Author, D. R. Reed, T. Hummel, S. D. Munger, J. E. Hayes, More than smell—COVID-19 is associated with severe impairment of smell, taste, and chemesthesis. *Chem. Senses* **45**, 609–622 (2020).

11. H. B. Stanley, V. Pereda-Campos, M. Mantel, C. Rouby, C. Daudé, P.-E. Aguera, L. Fornoni, T. Hummel, S. Weise, C. Mignot, I. Konstantinidis, K. Garefis, C. Ferdenzi, D. Pierron, M. Bensafi, Identification of the needs of individuals affected by COVID-19. *Commun. Med.* **4**, 83 (2024).

12. G. Besser, D. T. Liu, B. Renner, T. Hummel, C. A. Mueller, Olfactory implant: Demand for a future treatment option in patients with olfactory dysfunction. *Laryngoscope* **129**, 312–316 (2019).

13. A. Fournel, M. Mantel, M. Pinger, C. Manesse, R. Dubreuil, C. Herrier, T. Rousselle, T. Livache, M. Bensafi, An experimental investigation comparing a surface plasmon resonance imaging-based artificial nose with natural olfaction. *Sens. Actuators B Chem.* **320**, 128342 (2020).

14. K. Persaud, G. Dodd, Analysis of discrimination mechanisms in the mammalian olfactory system using a model nose. *Nature* **299**, 352–355 (1982).

15. M. Mantel, A. Fournel, I. Staedlé, A. Oelschlägel, J. Carro, R. Dubreuil, C. Herrier, T. Livache, A. Haehner, T. Hummel, J.-M. Roy, M. Bensafi, Using a bio-inspired surface resonance plasmon electronic nose for fundamental research on human olfaction. *Sens. Actuators B Chem.* **350**, 130846 (2022).

16. R. Haddad, A. Medhanie, Y. Roth, D. Harel, N. Sobel, Predicting odor pleasantness with an electronic nose. *PLOS Comput. Biol.* **6**, e1000740 (2010).

17. F. K. Che Harun, J. A. Covington, J. W. Gardner, Mimicking the biological olfactory system: A Portable electronic Mucosa. *IET Nanobiotechnol.* **6**, 45–51 (2012).

18. E. Aronsohn, Ueber elektrische geruchsempfindung. *Arch. Für Physiol.* **33**, 460–465 (1884). [On electrical olfactory perception].

19. E. H. Holbrook, S. V. Puram, R. B. See, A. G. Tripp, D. G. Nair, Induction of smell through transethmoid electrical stimulation of the olfactory bulb. *Int. Forum Allergy Rhinol.* **9**, 158–164 (2019).

20. G. Kumar, C. Juhász, S. Sood, E. Asano, Olfactory hallucinations elicited by electrical stimulation via subdural electrodes: Effects of direct stimulation of olfactory bulb and tract. *Epilepsy Behav.* **24**, 264–268 (2012).

21. A. Uziel, Neurophysiopathology of olfaction in man. *Sem. Hopitaux Organe Fonde Par Assoc. Enseign. Med. Hopitaux Paris* **54**, 351–352 (1978).

22. T. Weiss, S. Shushan, A. Ravia, A. Hahamy, L. Secundo, A. Weissbrod, A. Ben-Yakov, Y. Holtzman, S. Cohen-Atsmoni, Y. Roth, N. Sobel, From nose to brain: Un-sensed electrical currents applied in the nose alter activity in deep brain structures. *Cereb. Cortex* **26**, 4180–4191 (2016).

23. D. H. Coelho, L. D. Socolovsky, R. M. Costanzo, Activation of the rat olfactory bulb by direct ventral stimulation after nerve transection. *Int. Forum Allergy Rhinol.* **8**, 922–927 (2018).

24. N. Bérard, B. N. Landis, L. Legrand, R. Tyrand, F. Grouiller, S. Vulliémoz, S. Momjian, C. Boëx, Electrical stimulation of the medial orbitofrontal cortex in humans elicits pleasant olfactory perceptions. *Epilepsy Behav.* **114**, 107559 (2021).

25. M. R. Mercier, A.-S. Dubarry, F. Tadel, P. Avanzini, N. Axmacher, D. Cellier, M. D. Vecchio, L. S. Hamilton, D. Hermes, M. J. Kahana, R. T. Knight, A. Llorens, P. Megevand, L. Melloni, K. J. Miller, V. Piai, A. Puce, N. F. Ramsey, C. M. Schwiedrzik, S. E. Smith, A. Stolk, N. C. Swann, M. J. Vansteensel, B. Voytek, L. Wang, J.-P. Lachaux, R. Oostenveld, Advances in human intracranial electroencephalography research, guidelines and good practices. *Neuroimage* **260**, 119438 (2022).

26. M. Schulder, A. Mishra, A. Mammis, A. Horn, A. Boutet, P. Blomstedt, S. Chabardes, O. Flouty, A. M. Lozano, J. S. Neimat, F. Ponce, P. A. Starr, J. K. Krauss, M. Hariz, J. W. Chang, Advances in technical aspects of deep brain stimulation surgery. *Stereotact. Funct. Neurosurg.* **101**, 112–134 (2023).

27. S. Menzel, I. Konstantinidis, M. Valentini, P. Battaglia, M. Turri-Zanoni, G. Sileo, G. Monti, P. G. M. Castelnuovo, T. Hummel, A. Macchi, Surgical approaches for possible positions of an olfactory implant to stimulate the olfactory bulb. *ORL J. Otorhinolaryngol. Relat. Spec.* **85**, 253–263 (2023).

28. N. Gunder, P. Dörig, M. Witt, A. Welge-Lüssen, S. Menzel, T. Hummel, Future therapeutic strategies for olfactory disorders: Electrical stimulation, stem cell therapy, and transplantation of olfactory epithelium-an overview. *HNO* **71**, 35–43 (2023).

29. M. J. Proulx, D. J. Brown, A. Pasqualotto, P. Meijer, Multisensory perceptual learning and sensory substitution. *Neurosci. Biobehav. Rev.* **41**, 16–25 (2014).

30. M. Auvray, E. Myin, Perception with compensatory devices: From sensory substitution to sensorimotor extension. *Cognit. Sci.* **33**, 1036–1058 (2009).

31. P. Bach-y-Rita, S. W. Kercel, Sensory substitution and the human-machine interface. *Trends Cogn. Sci.* **7**, 541–546 (2003).

32. M. Ptito, S. M. Moesgaard, A. Gjedde, R. Kupers, Cross-modal plasticity revealed by electrotactile stimulation of the tongue in the congenitally blind. *Brain* **128**, 606–614 (2005).

33. A. C. Nau, M. C. Murphy, K. C. Chan, Use of sensory substitution devices as a model system for investigating cross-modal neuroplasticity in humans. *Neural Regen. Res.* **10**, 1717–1719 (2015).

34. G. Arnold, M. Auvray, Tactile recognition of visual stimuli: Specificity versus generalization of perceptual learning. *Vision Res.* **152**, 40–50 (2018).

35. J. Brooks, Á. Kristjánsson, R. Unnthorsson, "Sensory substitution: Visual information via haptics," in *Somatosensory Research Methods*, N. P. Holmes, Ed. (Springer, 2023), pp. 287–302.

36. P. Grant, M. Maeng, T. Arango, R. Hogle, J. Szlyk, W. Seiple, Performance of real-world functional tasks using an updated oral electronic vision device in persons blinded by trauma. *Optom. Vis. Sci.* **95**, 766–773 (2018).

37. M. Auvray, S. Hanneton, J. K. O'Regan, Learning to perceive with a visuo — Auditory substitution system: Localisation and object recognition with "The Voice". *Perception* **36**, 416–430 (2007).

38. D. Brown, T. Macpherson, J. Ward, Seeing with sound? Exploring different characteristics of a visual-to-auditory sensory substitution device. *Perception* **40**, 1120–1135 (2011).

39. MetaModal LLC - The vOICe; www.metamodal.com/.

40. P. L. Plaza, L. Renier, S. Rosemann, A. G. De Volder, J. P. Rauschecker, Sound-encoded faces activate the left fusiform face area in the early blind. *PLOS ONE* **18**, e0286512 (2023).

41. A. Neugebauer, K. Rifai, M. Getzlaff, S. Wahl, Navigation aid for blind persons by visual-to-auditory sensory substitution: A pilot study. *PLOS ONE* **15**, e0237344 (2020).

42. E. Striem-Amit, M. Guendelman, A. Amedi, 'Visual' acuity of the congenitally blind using visual-to-auditory sensory substitution. *PLOS ONE* **7**, e33136 (2012).

43. The vOICe - New Frontiers in Artificial Vision; www.seeingwithsound.com/.

44. Blind User Preferences for Sensory Substitution Devices (SCHI Lab) (2017); www.youtube.com/watch?v=ha0aL2VAwCw.

45. I. Kohler, M. V. Perrotta, T. Ferreira, D. M. Eagleman, Cross-modal sensory boosting to improve high-frequency hearing loss: Device development and validation. *JMIRx Med.* **5**, e49969 (2024).

46. M. Laska, H. Distel, R. Hudson, Trigeminal perception of odorant quality in congenitally anosmic subjects. *Chem. Senses* **22**, 447–456 (1997).

47. R.-P. Filiou, F. Lepore, B. Bryant, J. N. Lundström, J. Frasnelli, Perception of trigeminal mixtures. *Chem. Senses* **40**, 61–69 (2015).

48. K. Aoyama, N. Miyamoto, S. Sakurai, H. Iizuka, M. Mizukami, M. Furukawa, T. Maeda, H. Ando, Electrical generation of intranasal irritating chemosensation. *IEEE Access* **9**, 106714–106724 (2021).

49. T. Hummel, J. Frasnelli, "Chapter 8 - The intranasal trigeminal system," in *Handbook of Clinical Neurology*, R. L. Doty, Ed. (Elsevier, 2019), vol. 164, pp. 119–134; www.sciencedirect.com/science/article/pii/B9780444638557000083.

50. M. Q. Nguyen, Y. Wu, L. S. Bonilla, L. J. von Buchholtz, N. J. P. Ryba, Diversity amongst trigeminal neurons revealed by high throughput single cell sequencing. *PLOS ONE* **12**, e0185543 (2017).

51. M. Scheibe, T. Zahnert, T. Hummel, Topographical differences in the trigeminal sensitivity of the human nasal mucosa. *Neuroreport* **17**, 1417–1420 (2006).

52. R. L. Doty, W. E. Brugger, P. C. Jurs, M. A. Orndorff, P. J. Snyder, L. D. Lowry, Intranasal trigeminal stimulation from odorous volatiles: Psychometric responses from anosmic and normal humans. *Physiol. Behav.* **20**, 175–185 (1978).

53. R. L. Doty, Treatments for smell and taste disorders: A critical review. *Handb. Clin. Neurol.* **164**, 455–479 (2019).

54. M. Pinger, J. Draf, T. Lakner, C. Ferdenzi, C. Rouby, T. Hummel, M. Bensafi, Perceived utility of electronic noses in patients with loss of smell. *Eur. Arch. Otorhinolaryngol.* **278**, 2155–2156 (2021).

55. P. Bach-Y-Rita, C. C. Collins, F. A. Saunders, B. White, L. Scadden, Vision substitution by tactile image projection. *Nature* **221**, 963–964 (1969).

56. J. Kilian, A. Neugebauer, L. Scherffig, S. Wahl, The unfolding space glove: A wearable spatio-visual to haptic sensory substitution device for blind people. *Sensors* **22**, 1859 (2022).

57. D.-R. Chebat, F. C. Schneider, M. Ptito, Spatial competence and brain plasticity in congenital blindness via sensory substitution devices. *Front. Neurosci.* **14**, 815 (2020).

58. M. D. Fletcher, E. Akis, C. A. Verschuur, S. W. Perry, Improved tactile speech perception using audio-to-tactile sensory substitution with formant frequency focusing. *Sci. Rep.* **14**, 4889 (2024).

59. V. D. Ruiz-Stovel, A. A. González-Garrido, F. R. Gómez-Velázquez, G. B. Gallardo-Moreno, E. R. Villuendas-González, C. A. Soto-Nava, Assessment of audio-tactile sensory substitution training in participants with profound deafness using the event-related potential technique. *J. Vis. Exp.*, e64266 (2022).

60. J. A. Gottfried, J. O'Doherty, R. J. Dolan, Appetitive and aversive olfactory learning in humans studied using event-related functional magnetic resonance imaging. *J. Neurosci.* **22**, 10829–10837 (2002).

61. R. Vincis, A. Fontanini, Associative learning changes cross-modal representations in the gustatory cortex. *eLife* **5**, e16420 (2016).

62. C. C. Licon, G. Bosc, M. Sabri, M. Mantel, A. Fournel, C. Bushdid, J. Golebiowski, C. Robardet, M. Plantevit, M. Kaytoue, M. Bensafi, Chemical features mining provides new descriptive structure-odor relationships. *PLOS Comput. Biol.* **15**, e1006945 (2019).

63. R. M. Khan, C.-H. Luk, A. Flinker, A. Aggarwal, H. Lapid, R. Haddad, N. Sobel, Predicting odor pleasantness from odorant structure: Pleasantness as a reflection of the physical world. *J. Neurosci.* **27**, 10015–10023 (2007).

64. M. Bensafi, T. Livache, C. Herrier, C. Ferdenzi, R. Dubreuil, Procédés et systèmes de substitution sensorielle de l'odorat chez un sujet (2024); https://patents.google.com/patent/WO2024200803A1/fr. [Methods and systems for the sensory substitution of smell in a subject].

65. C. S. Green, D. Bavelier, Exercising your brain: A review of human brain plasticity and training-induced learning. *Psychol. Aging* **23**, 692–701 (2008).

66. J. Mishra, E. de Villers-Sidani, M. Merzenich, A. Gazzaley, Adaptive training diminishes distractibility in aging across species. *Neuron* **84**, 1091–1103 (2014).

67. O. Collignon, F. Champoux, P. Voss, F. Lepore, Sensory rehabilitation in the plastic brain. *Prog. Brain Res.* **191**, 211–231 (2011).

68. I. Konstantinidis, E. Tsakiropoulou, P. Bekiaridou, C. Kazantzidou, J. Constantinidis, Use of olfactory training in post-traumatic and postinfectious olfactory dysfunction. *Laryngoscope* **123**, E85–E90 (2013).

69. M. Pieniak, A. Oleszkiewicz, V. Avaro, F. Calegari, T. Hummel, Olfactory training – Thirteen years of research reviewed. *Neurosci. Biobehav. Rev.* **141**, 104853 (2022).

70. T. Hummel, K. Rissom, J. Reden, A. Hähner, M. Weidenbecher, K. Hüttenbrink, Effects of olfactory training in patients with olfactory loss. *Laryngoscope* **119**, 496–499 (2009).

71. A. Fournel, C. Sezille, C. C. Licon, C. Sinding, J. Gerber, C. Ferdenzi, T. Hummel, M. Bensafi, Learning to name smells increases activity in heteromodal semantic areas. *Hum. Brain Mapp.* **38**, 5958–5969 (2017).

72. J. B. Nielsen, M. Willerslev-Olsen, L. Christiansen, J. Lundbye-Jensen, J. Lorentzen, Science-based neurorehabilitation: Recommendations for neurorehabilitation from basic Science. *J. Mot. Behav.* **47**, 7–17 (2015).

73. J. Mainland, N. Sobel, The sniff is part of the olfactory percept. *Chem. Senses* **31**, 181–196 (2006).

74. F. Genovese, H. G. Bauersachs, I. Gräßer, J. Kupke, L. Magin, P. Daiber, J. Nakajima, F. Möhrlen, K. Messlinger, S. Frings, Possible role of calcitonin gene-related peptide in trigeminal modulation of glomerular microcircuits of the rodent olfactory bulb. *Eur. J. Neurosci.* **45**, 587–600 (2017).

75. M. Maurer, N. Papotto, J. Sertel-Nakajima, M. Schueler, R. De Col, F. Möhrlen, K. Messlinger, S. Frings, R. W. Carr, Photoactivation of olfactory sensory neurons does not affect action potential conduction in individual trigeminal sensory axons innervating the rodent nasal cavity. *PLOS ONE* **14**, e0211175 (2019).

76. M. L. Schaefer, B. Böttger, W. L. Silver, T. E. Finger, Trigeminal collaterals in the nasal epithelium and olfactory bulb: A potential route for direct modulation of olfactory information by trigeminal stimuli. *J. Comp. Neurol.* **444**, 221–226 (2002).

77. J. A. Boyle, M. Heinke, J. Gerber, J. Frasnelli, T. Hummel, Cerebral activation to intranasal chemosensory trigeminal stimulation. *Chem. Senses* **32**, 343–353 (2007).

78. J. Frasnelli, T. Hummel, J. Berg, G. Huang, R. L. Doty, Intranasal localizability of odorants: Influence of stimulus volume. *Chem. Senses* **36**, 405–410 (2011).

79. J. Brooks, S.-Y. Teng, J. Wen, R. Nith, J. Nishida, P. Lopes, "Stereo-smell via electrical trigeminal stimulation," in *Proceedings of the 2021 CHI Conference on Human Factors in Computing Systems* (ACM, 2021), pp. 1–13; https://dl.acm.org/doi/10.1145/3411764.3445300.

80. C. C. Licon, C. Manesse, M. Dantec, A. Fournel, M. Bensafi, Pleasantness and trigeminal sensations as salient dimensions in organizing the semantic and physiological spaces of odors. *Sci. Rep.* **8**, 8444 (2018).

81. A. Fournel, C. Ferdenzi, C. Sezille, C. Rouby, M. Bensafi, Multidimensional representation of odors in the human olfactory cortex. *Hum. Brain Mapp.* **37**, 2161–2172 (2016).

82. R. B. Zajonc, Attitudinal effects of mere exposure. *J. Pers. Soc. Psychol.* **9**, 1–27 (1968).

83. T. Hummel, G. Kobal, H. Gudziol, A. Mackay-Sim, Normative data for the "Sniffin' Sticks" including tests of odor identification, odor discrimination, and olfactory thresholds: An upgrade based on a group of more than 3,000 subjects. *Eur. Arch. Otorhinolaryngol.* **264**, 237–243 (2007).

84. jamovi, The jamovi project, version 2.3 (2022); www.jamovi.org.

85. R Core Team, R: A Language and environment for statistical computing, version 4.1 (2021) (R packages retrieved from MRAN snapshot 2022-01-01); https://cran.r-project.org.

86. K. Garefis, S. Weise, P. Hanslik, C. Mignot, H. B. Stanley, M. Fieux, C. Ferdenzi, E. Tsakiropoulou, C. Lipp, A. Bertsch, M. Bensafi, I. Konstantinidis, T. Hummel, Electrical stimulation of trigeminal nerve at the anterior nasal septum in healthy individuals and patients with olfactory dysfunction. *Int. Forum Allergy Rhinol.*, 10.1002/alr.70003 (2025).

87. C. Lipp, E. Glushkov, H. B. Stanley, C. Ferdenzi, M. Fieux, A. Bertsch, J. Brugger, M. Bensafi, "Stimulation of the nasal cavity using flexible PCB electrodes," in *Proceedings XXXV EUROSENSORS Conference* (MDPI, 2024), p. 231.

88. J. Frasnelli, T. Hummel, Age-related decline of intranasal trigeminal sensitivity: Is it a peripheral event? *Brain Res.* **987**, 201–206 (2003).
